# Supplementary material for: RIPK1‐mediated immunogenic cell death promotes anti‐tumour immunity against soft‐tissue sarcoma
Source: EMBO Mol Med. 2020 May 18;12(6):e10979. doi: 10.15252/emmm.201910979 (PMC7278545; doi:10.15252/emmm.201910979)
Supplement: Supplementary file 1 — Appendix [file EMMM-12-e10979-s001.docx]

**RIPK1-mediated immunogenic cell death promotes anti-tumour immunity against soft-tissue sarcoma**

**By Smith, Jamal, Dayal *et al.***

Table of Contents

Table S1. Exact P-values and Statistical tests used in Figures 1-6 and Figures EV1-EV6. 2

**P-value calculations………………………………………………………………5**

## Table S1. Exact P-values and Statistical tests used in Figures 1-6 and Figures EV1-EV6.

| **Figure** | **Statistical Test** | **Comparison** | **P value** |
| --- | --- | --- | --- |
| Fig. 1A | Unpaired *t-test* | TNF/Mel vs TNF/Mel/SM at 24 hrs | 0.00000081 |
|  | Unpaired *t-test* | TNF/Mel vs TNF/Mel/SM at 48 hrs | 0.0000558 |
| Fig. 1D | Unpaired *t-test* | TNF/Mel vs TNF/Mel/SM | 0.00000005 |
| Fig. 1E | One-way *Anova* | Ctrl TNF/Mel/SM vs *Ripk1^-/-^* guide 1 TNF/Mel/SM at 18 hrs | 0.00002290 |
|  | One-way *Anova* | Ctrl TNF/Mel/SM vs *Ripk1^-/-^* guide 2 TNF/Mel/SM at 18 hrs | 0.00000324 |
|  | One-way *Anova* | Ctrl TNF/Mel/SM vs *Casp-8^-/-^* guide 1 TNF/Mel/SM at 18 hrs | 0.00000252 |
|  | One-way *Anova* | Ctrl TNF/Mel/SM vs *Casp-8^-/-^* guide 2 TNF/Mel/SM at 18 hrs | 0.00003854 |
|  | One-way *Anova* | Ctrl TNF/Mel/SM vs *Ripk1^-/-^* guide 1 TNF/Mel/SM at 48 hrs | 0.5235 |
|  | One-way *Anova* | Ctrl TNF/Mel/SM vs *Ripk1^-/-^* guide 2 TNF/Mel/SM at 48 hrs | 0.3001 |
|  | One-way *Anova* | Ctrl TNF/Mel/SM vs *Casp-8^-/-^* guide 1 TNF/Mel/SM at 48 hrs | 0.2735 |
|  | One-way *Anova* | Ctrl TNF/Mel/SM vs *Casp-8^-/-^* guide 2 TNF/Mel/SM at 48 hrs | 0.1942 |
| Fig. 2B | Unpaired *t-test* | Untreated vs TNF/SM | 0.0000000017 |
| Fig. 2C | One-way *Anova* | siCtrl TNF/SM vs *siRIPK1* TNF/SM | 0.0000011951 |
|  | One-way *Anova* | siCtrl TNF/SM vs *siCASP-8* TNF/SM | 0.0000006932 |
| Fig. 2E | Unpaired *t-test* | Untreated vs TNF/SM | 0.0000012750 |
| Fig. 2F | Unpaired *t-test* | siCtrl TNF/SM vs *siRIPK1* TNF/SM | 0.0000014227 |
|  | Unpaired *t-test* | siCtrl TNF/SM vs *siCASP-8* TNF/SM | 0.0000034878 |
| Fig. 2G | Unpaired *t-test* | Untreated vs TNF/SM | 0.0000098180 |
| Fig. 2H | Unpaired *t-test* | Untreated vs TNF/SM | 0.0000004521 |
| Fig. 2I | Unpaired *t-test* | Untreated vs SM | 0.0000014303 |
|  | Unpaired *t-test* | Untreated vs TNF/SM | 0.0000005173 |
|  | Unpaired *t-test* | SM vs TNF/SM | 0.0003881713 |
| Fig. 2J | Unpaired *t-test* | Untreated vs TNF/SM | 0.0008 |
| Fig. 2K | Unpaired *t-test* | Untreated vs TNF/SM | 0.0002 |
| Fig. 2L | One-way *Anova* | Untreated vs TNF/SM | 0.0000000104 |
|  | One-way *Anova* | TNF/SM vs TNF/SM/zVAD | 0.0000070357 |
| Fig. 2M | One-way *Anova* | Untreated vs TNF/SM | 0.0000000002 |
|  | One-way *Anova* | TNF/SM vs TNF/SM/zVAD | 0.0000000378 |
| Fig. 2N | One-way *Anova* | Untreated vs TNF/SM | 0.0000000226 |
|  | One-way *Anova* | TNF/SM vs TNF/SM/zVAD | 0.0000024526 |
| Fig. 3A | Unpaired *t-test* | ILP-Control vs ILP-TNF/Mel | 0.0001 |
|  | Unpaired *t-test* | ILP-TNF/Mel vs ILP-TNF/Mel/SM | 0.03 |
| Fig. 3B | *Log-rank test* | ILP-Control vs ILP-TNF/Mel | 0.0048 |
|  | *Log-rank test* | ILP-TNF/Mel vs ILP-TNF/Mel/SM | 0.0049 |
| Fig. 3C | Unpaired *t-test* | ILP-TNF/Mel vs ILP-TNF/Mel/SM | 0.0034 |
| Fig. 3E | One-way *Anova* | Parental TNF/Mel/SM vs tumour line 124 TNF/Mel/SM | 0.1762 |
|  | One-way *Anova* | Parental TNF/Mel/SM vs tumour line 133 TNF/Mel/SM | 0.7676 |
|  | One-way *Anova* | Parental TNF/Mel/SM vs tumour line 136 TNF/Mel/SM | 0.8351 |
| Fig. 3F | One-way *Anova* | Ctrl SM/RIBOXXOL vs  *Ripk1^-/-^* guide 1 SM/RIBOXXOL | 0.0000000606 |
|  |  | Ctrl SM/RIBOXXOL vs  *Ripk1^-/-^* guide 2 SM/RIBOXXOL | 0.0000000067 |
|  |  | Ctrl SM/RIBOXXOL vs  *Casp-8^-/-^* guide 1 | 0.0000000389 |
|  |  | Ctrl SM/RIBOXXOL vs  *Casp-8^-/-^* guide 2 SM/RIBOXXOL | 0.0000001809 |
| Fig. 4B | One-way *Anova* | Control vs TNF/Mel/SM | 0.0281 |
| Fig. 4C | One-way *Anova* | Control vs TNF/Mel/SM | 0.0403 |
| Fig. 4D | One-way *Anova* | Control vs TNF/Mel | 0.0216 |
|  | One-way *Anova* | TNF/Mel vs TNF/Mel/SM | 0.0081 |
| Fig. 4E | One-way *Anova* | Control vs TNF/Mel/SM | 0.0027 |
|  | One-way *Anova* | TNF/Mel vs TNF/Mel/SM | 0.0113 |
| Fig. 4F | One-way *Anova* | Control vs TNF/Mel/SM | 0.0031 |
|  | One-way *Anova* | TNF/Mel vs TNF/Mel/SM | 0.0010 |
| Fig. 4G | One-way *Anova* | Control vs TNF/Mel/SM | > 0.0001 |
|  | One-way *Anova* | TNF/Mel vs TNF/Mel/SM | 0.0003 |
| Fig. 4H | One-way *Anova* | Control vs TNF/Mel/SM | 0.0010 |
|  | One-way *Anova* | TNF/Mel vs TNF/Mel/SM | 0.0007 |
| Fig. 5B | *Log-rank test* | ILP-TNF/Mel/SM vs ILP-TNF/Mel/SM/anti-CTLA-4 | 0.0253 |
| Fig. 5C | One-way *Anova* | ILP-TNF/Mel/SM vs ILP-TNF/Mel/SM/anti-CTLA-4 | 0.0344 |
| Fig. 5D | One-way *Anova* | ILP-TNF/Mel/SM vs ILP-TNF/Mel/SM/anti-CTLA-4 | 0.0270 |
|  | One-way *Anova* | ILP-TNF/Mel/SM/anti-PD-1 vs ILP-TNF/Mel/SM/anti-CTLA-4 | 0.0197 |
| Fig. 5E | One-way *Anova* | ILP-TNF/Mel/SM and ILP-TNF/Mel/SM/anti-CTLA-4 vs ILP-TNF/Mel/SM/anti-PD-1 | 0.0183 |
| Fig. 6B | Unpaired *t-test* | ILP-TNF/Mel vs ILP-TNF/Mel/SM | 0.0000002129 |
| Fig. 6E | *Log-rank test* | ILP-TNF/Mel vs ILP-TNF/Mel/SM | 0.0259 |
| EV1B | Unpaired *t-test* | Untreated vs TNF/Mel/SM | 0.0000006393 |
|  | Unpaired *t-test* | TNF/Mel/SM vs TNF/Mel/SM/RIPK1i | 0.0000026685 |
|  | Unpaired *t-test* | TNF/Mel vs TNF/Mel/RIPK1i | 0.0628 |
| EV1E | Unpaired *t-test* | Mel WT vs Mel BCL2 | 0.0000028314 |
|  | Unpaired *t-test* | TNF/Mel WT vs TNF/Mel BCL2 | 0.0000002479 |
| EV2D | One-way *Anova* | Untreated vs SM | 0.0000013833 |
|  | One-way *Anova* | Untreated vs TNF/SM | 0.0000000013 |
| EV2F | Unpaired *t-test* | Untreated vs TNF/SM | 0.0000000038 |
| EV2H | Unpaired *t-test* | Untreated vs TNF/SM | 0.0000007280 |
| EV2I | Unpaired *t-test* | Untreated vs TNF/SM | 0.0029 |
| EV2J | Unpaired *t-test* | Untreated vs TNF/SM | 0.0008 |
| EV2K | Unpaired *t-test* | Untreated vs TNF/SM | 0.0009 |
| EV2L | One-way *Anova* | Untreated vs SM | 0.0005 |
|  | One-way *Anova* | SM vs TNF/SM | 0.0005 |
| EV2M | One-way *Anova* | Untreated vs TNF/SM | > 0.0001 |
|  | One-way *Anova* | TNF/SM vs TNF/SM/zVAD | 0.0001 |
| EV2N | One-way *Anova* | Untreated vs TNF/SM | > 0.0001 |
|  | One-way *Anova* | TNF/SM vs TNF/SM/zVAD | 0.0078 |

**P-value calculations:**

|  |  | =T.DIST.2T(111.2, 4) |
| --- | --- | --- |
| Fig 1A 24hrs | t=52.17 df=4 | 0.00000081 |
|  |  |  |
| Fig1A 48hrs | t=18.01 df=4 | 0.00005588 |
|  |  |  |
| Fig 1D | t=105 df=4 | 0.00000005 |
|  |  |  |
| Fig 1E |  |  |
| cnt v ripk1 g1 | t=22.55 df=4 | 0.00002290 |
|  |  |  |
| cnt v ripk1 g2 | t=36.84 df=4 | 0.00000324 |
|  |  |  |
| cnt v casp8 g1 | t=39.25 df=4 | 0.00000252 |
|  |  |  |
| cnt v casp8 g2 | t=19.78 df=4 | 0.00003854 |
|  |  |  |
| Fig 2B | t=243.3 df=4 | 0.0000000017 |
|  |  |  |
| Fig 2C | t=47.3 df=4 | 0.0000011951 |
|  |  |  |
|  | t=54.21 df=4 | 0.0000006932 |
|  |  |  |
| Fig 2E | t=46.54 df=4 | 0.0000012750 |
|  |  |  |
| Fig. 2F | t=45.28 df=4 | 0.0000014227 |
|  |  |  |
|  | t=36.17 df=4 | 0.0000034878 |
|  |  |  |
| Fig. 2G | t=27.9 df=4 | 0.0000098180 |
|  |  |  |
| Fig. 2H | t=60.33 df=4 | 0.0000004521 |
|  |  |  |
| Fig. 2I | t=45.22 df=4 | 0.0000014303 |
|  |  |  |
|  | t=58.33 df=4 | 0.0000005173 |
|  |  |  |
|  | t=11 df=4 | 0.0003881713 |
|  |  |  |
| Fig. 2J | t=34.3 df=2 | 0.0008489038 |
|  |  |  |
| Fig. 2L | t=17.01 df=10 | 0.0000000104 |
|  |  |  |
|  | t=8.481 df=10 | 0.0000070357 |
|  |  |  |
| Fig. 2M | t=24.91 df=10 | 0.0000000002 |
|  |  |  |
|  | t=14.88 df=10 | 0.0000000378 |
|  |  |  |
|  | t=15.692 df=10 | 0.0000000226 |
|  |  |  |
|  | t=9.536 df=10 | 0.0000024526 |
|  |  |  |
| Fig. 3F | t=99.72 df=4 | 0.0000000606 |
|  |  |  |
|  | t=172.8 df=4 | 0.0000000067 |
|  |  |  |
|  | t=111.4 df=4 | 0.0000000389 |
|  |  |  |
|  | t=75.87 df=4 | 0.0000001809 |
|  |  |  |
| Fig. 6B | t=12.41 df=10 | 0.0000002129 |
|  |  |  |
| EV1 | t=55.32 df=4 | 0.0000006393 |
|  |  |  |
|  | t=38.68 df=4 | 0.0000026685 |
|  |  |  |
|  | t=38.11 df=4 | 0.0000028314 |
|  |  |  |
|  | t=70.12 df=4 | 0.0000002479 |
|  |  |  |
| EV2D | t=45.6 df=4 | 0.0000013833 |
|  |  |  |
|  | t=260.9 df=4 | 0.0000000013 |
|  |  |  |
| EV2F | t=199.1 df=4 | 0.0000000038 |
|  |  |  |
| EV2H | t=53.55 df=4 | 0.0000007280 |
|  |  |  |
| EV2I |  |  |
|  |  |  |
| EV2J |  |  |
|  |  |  |
| EV2K |  |  |
|  |  |  |
| EV2L |  |  |
|  |  |  |
| EV2M |  |  |
|  |  |  |
| EV2N |  |  |
