# Supplementary material for: RIPK1‐mediated immunogenic cell death promotes anti‐tumour immunity against soft‐tissue sarcoma
Source: EMBO Mol Med. 2020 May 18;12(6):e10979. doi: 10.15252/emmm.201910979 (PMC7278545; doi:10.15252/emmm.201910979)
Supplement: Supplementary file 2 — Expanded View Figures PDF [file EMMM-12-e10979-s002.pdf]

Expanded View Figures

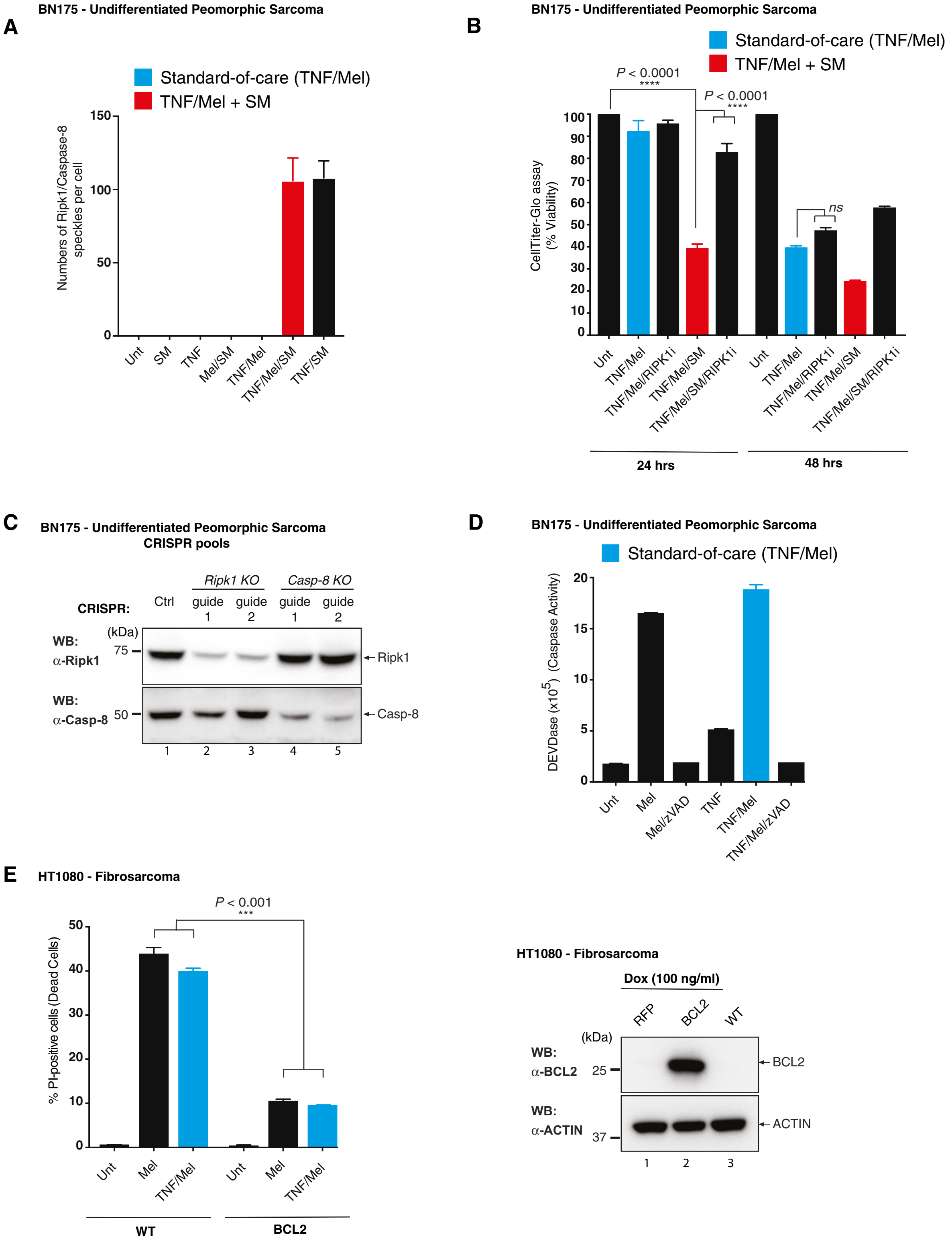

Figure EV1.

© 2020 The Authors

Figure EV1. TNF/Mel/SM-induced cell death.

A *In situ* PLA detection of Ripk1::Casp-8 in BN175 cells. Quantification of Ripk1/Casp-8 speckles per cell from PLA in Fig 1C (n = 2 biological replicates). Ripk1/Casp-8 speckles intensity was normalised to each biological replicate for background correction prior to being represented as one aggregated graph. DMSO (Unt), zVAD (10 μM), Mel (3.3 μM), TNF (10 ng/ml) and SM (100 ng/ml). SM represents SM-164. Error bars represent SD.

B Cell viability analysis using CellTiter-Glo of BN175 CRISPR/Cas9 *Ripk1* and *Casp-8* knockouts treated with the indicated drugs for 24 and 48 h (n = 3 biological replicates). DMSO (Unt), RIPK1i (100 nM), Mel (3.3 μM), TNF (10 ng/ml) and SM (100 ng/ml). SM represents SM-164. Error bars represent SD. Statistical analysis was performed with an unpaired t-test, ****$P \le 0.0001$, ns = not significant. Exact P values are shown in Appendix Table S1.

C Western blot analysis of BN175 CRISPR/Cas9 *Ripk1* and *Casp-8* knockouts.

D DEVDase activity assay of BN175 cells treated with the indicated drugs for 24 h (n = 2 biological replicates). DMSO (Unt), TNF (10 ng/ml), Mel (3.3 μM) and zVAD (3.3 μM).

E Cell death analysis measured by Celigo of PI-positive HT1080 cells, and cells were treated with the indicated agents for 48 h. (n = 3 biological replicates). DMSO (Unt), zVAD (10 μM), TNF (10 ng/ml) and Mel (50 μM). Error bars represent SD and an unpaired t-test was performed, ***$P \le 0.001$. Western blot analysis of HT1080 and HT1080 $BCL2^{-/-}$ treated with Dox (100 ng/ml) to induce BCL2 or RFP expression. Exact P values are shown in Appendix Table S1.

Figure EV2. TNF and SM sensitise a panel of human extremity malignancies cell lines to caspase-dependent cell death.

A Immunoprecipitation of TNFR1 signalling complexes, depicting the association of indicated components. HT1080 cells were treated with FLAG-hTNF (0.8 μg/ml) for the indicated time points, followed by FLAG immunoprecipitation and Western blot analysis. (n = 3 biological replicates).

B, C DEVDase (caspase) activity assay of HT1080 (A) cells and SW-872 (B) cells treated with DMSO (Unt), zVAD-FMK (10 μM), TNF (10 ng/ml) or SM (100 ng/ml) for 6 h (n = 3 biological replicates). SM represents SM-164. Error bars represent SD.

D Cell viability analysis using CellTiter-Glo of SW-872 cells treated with the indicated drugs for 24 h (n = 3 biological replicates). DMSO (Unt), zVAD-FMK (10 μM), TNF (10 ng/ml) or SM (100 ng/ml). SM represents SM-164. Error bars represent SD. Statistical analysis was performed with a one-way *ANOVA*, ****$P \le 0.0001$. Exact P values are shown in Appendix Table S1.

E DEVDase (caspase) activity assay of A375 cells treated with the indicated agents for 6 h (n = 3 biological replicates). DMSO (Unt), zVAD-FMK (10 μM), TNF (10 ng/ml) or SM (100 ng/ml). SM represents SM-164. Error bars represent SD.

F Cell viability analysis using CellTiter-Glo of A375 cells treated with the indicated drugs for 24 h (n = 3 biological replicates). DMSO (Unt), zVAD-FMK (10 μM), TNF (10 ng/ml) or SM (100 ng/ml). SM represents SM-164. Error bars represent SD. Statistical analysis was performed with an unpaired t-test, ****$P \le 0.0001$. Exact P values are shown in Appendix Table S1.

G DEVDase (caspase) activity assay of DO4 cells treated with the indicated agents for 6 h (n = 3 biological replicates). DMSO (Unt), zVAD-FMK (10 μM), TNF (10 ng/ml) or SM (100 ng/ml). SM represents SM-164. Error bars represent SD.

H Cell viability analysis using CellTiter-Glo of DO4 cells treated with the indicated agents for 24 h (n = 3 biological replicates). DMSO (Unt), zVAD-FMK (10 μM), TNF (10 ng/ml) or SM (100 ng/ml). SM represents SM-164. Error bars represent SD. Statistical analysis was performed with an unpaired t-test, ****$P \le 0.0001$. Exact P values are shown in Appendix Table S1.

I–L Cell death analysis by Celigo of PI-positive HS-ES-2M (I), HS-ES-1(J), SW-982 (K) and SCC-12 (L) cells. Cells were treated with the indicated agents for 48 h (HS-ES-2M, HS-ES-1 and SW-982) or 24 h (SCC-12). DMSO (Unt), zVAD (10 μM), TNF (100 ng/ml) and SM (100 ng/ml) SM represents SM-164. Error bars represent SD. Displayed are representative results from n = 3, and statistical analysis was performed with one-way *ANOVA*, ***$P \le 0.001$. Exact P values are shown in Appendix Table S1.

M, N Cell Death analysis by IncuCyte Zoom of CellTox Green-positive MET4 (M) and T1 (N) cells. Cells were treated with the indicated agents for 40 h (MET4) or 60 h (T1). DMSO (Unt), zVAD (10 μM), TNF (10 ng/ml) and SM (100 ng/ml) SM represents SM-164. Error bars represent SD. Displayed are representative results from n = 6, and statistical analysis was performed with a one-way *ANOVA*, **$P \le 0.001$, ***$P \le 0.001$, ****$P \le 0.0001$. Exact P values are shown in Appendix Table S1.

© 2020 The Authors

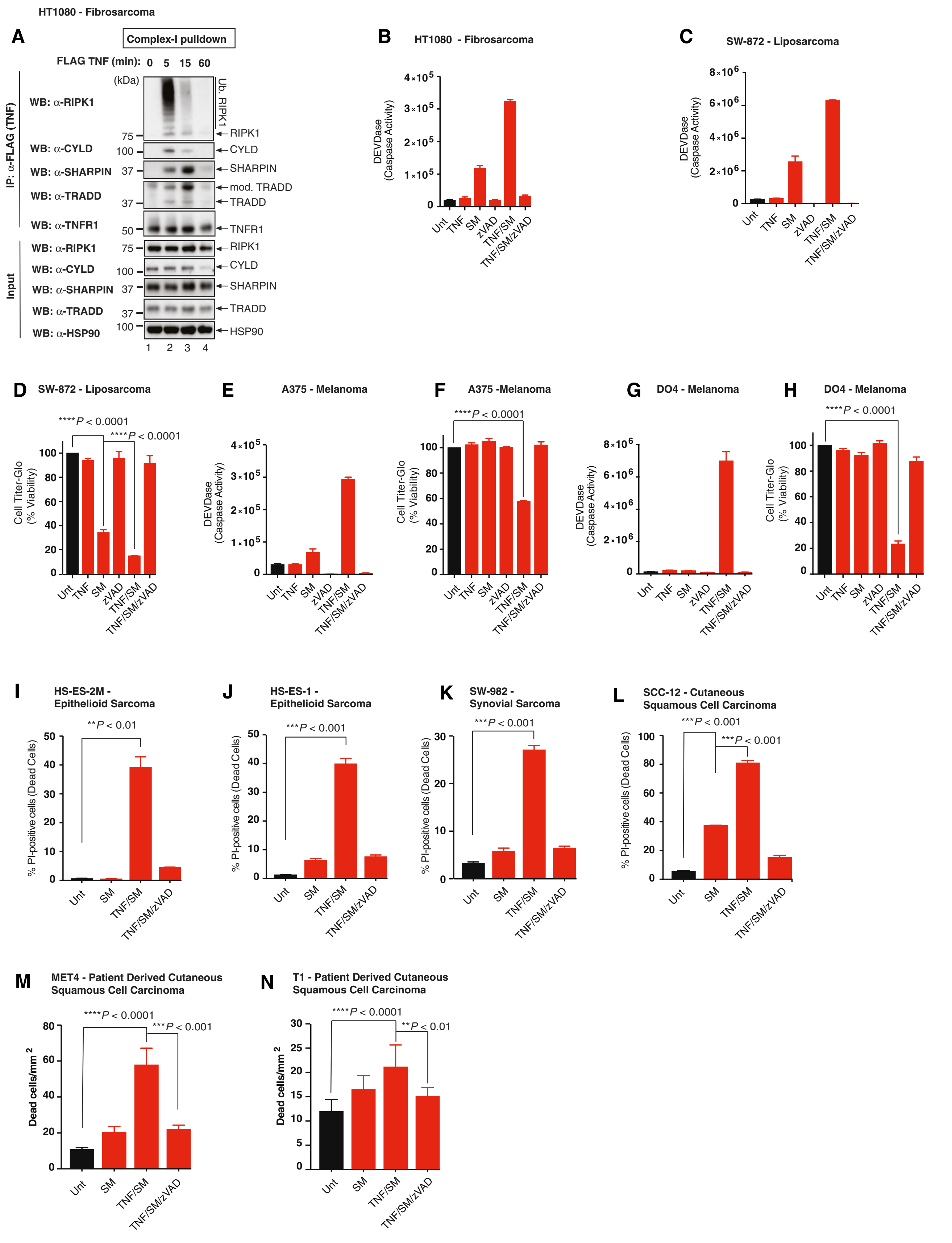

Figure EV2.

 © 2020 The Authors

Figure EV3. ILP-TNF/Mel/SM treatment results in increased locoregional toxicity compared with standard-of-care ILP.

A Cell viability analysis using CellTiter-Glo BN175 cells treated with indicated agents for 24 h (n = 3 biological replicates). SM represents Birinapant. Error bars represent SD. Statistical analysis was performed with a one-way *ANOVA*, ****$P \leq 0.0001$. Exact P values are shown in Appendix Table S1.

B–E Individual tumour growth curves following ILP treatment with (A) vehicle control, (B) TNF/Mel, (C) SM or (D) TNF/Mel/SM (n = 6 per cohort).

F Five days post-treatment, TNF/Mel/SM-treated animals developed grade III reactions. Locoregional toxicity observed in animals treated with TNF/Mel/SM gradually resolved 2 weeks post-treatment (n = 6 per cohort). SM represents Birinapant.

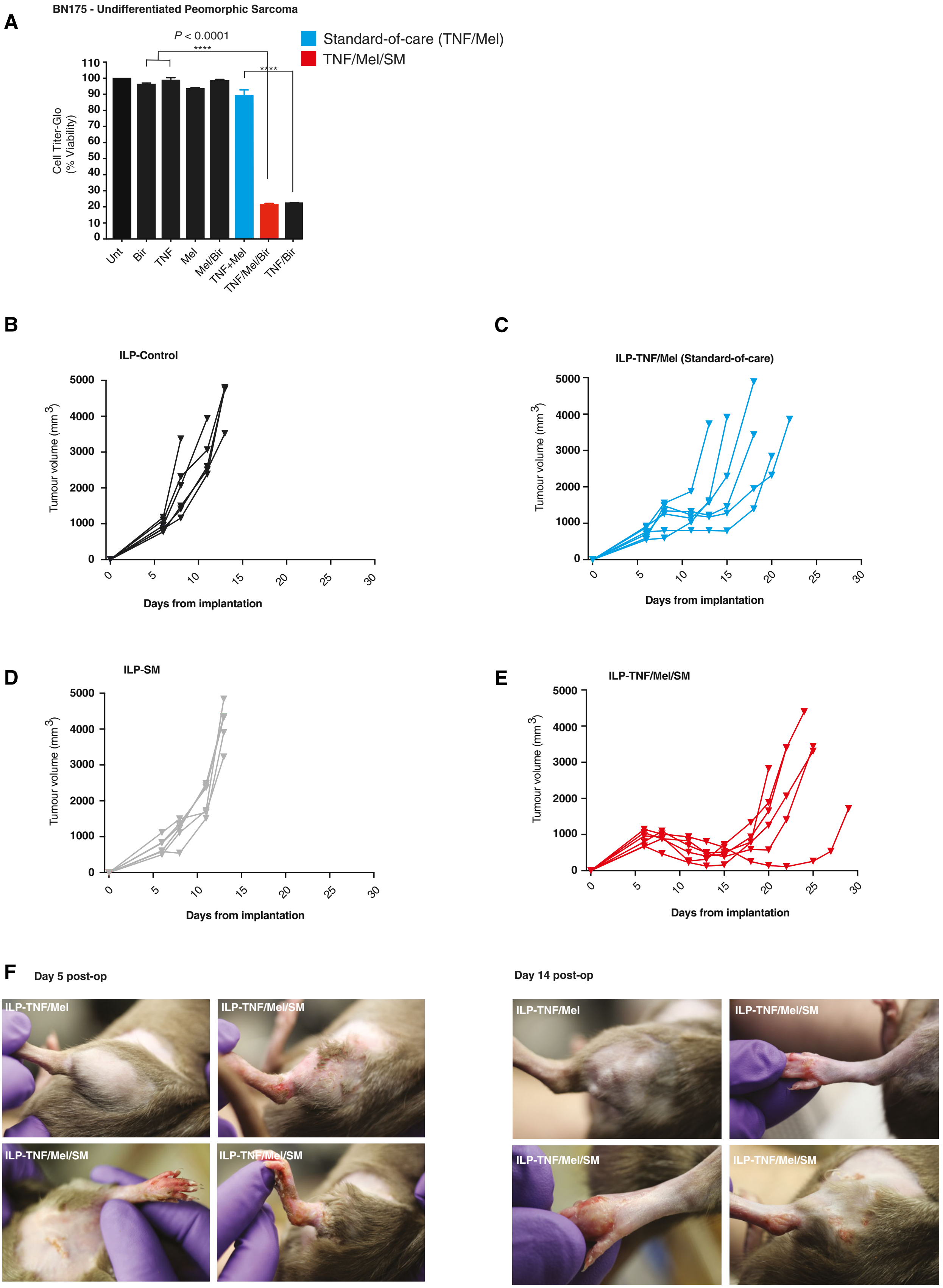

Figure EV3.

© 2020 The Authors

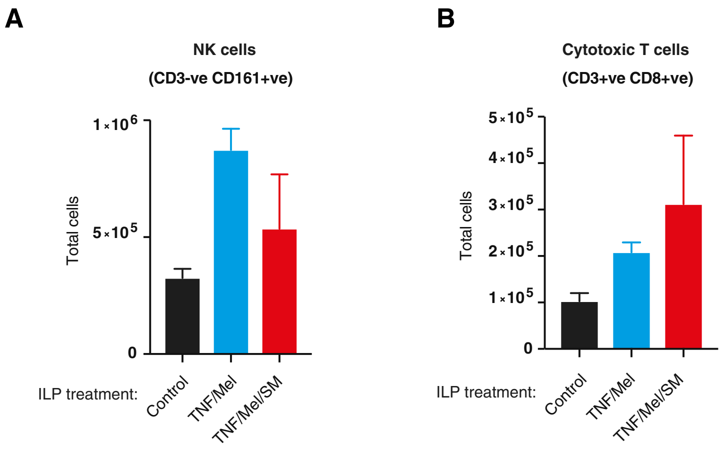

C

	Mean Granzyme B of CD8+ T cells
ILP-TNF/Mel	130.5
ILP-TNF/Mel/SM	396.7

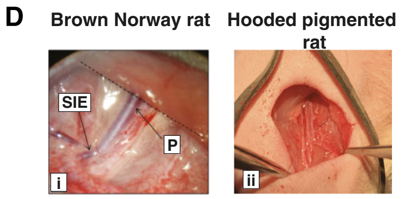

Figure EV4. ILP-TNF/Mel/SM treatment results in an increase in total numbers of infiltrating $CD8^+$ T cells.

A–C Flow cytometry analysis of tumours collected on Day 10 following treatment (*n* = 3 animals per group). (A) ILP-mediated treatment with TNF/Mel/SM has little impact on the number of total NK cells in the tumour. (B) Treatment with TNF/Mel/SM increased the total numbers of $CD3^+CD8^+$ cytotoxic T cells. (C) Mean Granzyme B expression levels in cytotoxic T cells.

D Microscopic view of Brown Norway femoral vessels (i), indicating site of origin of profunda femoris vessels (P) and superficial inferior epigastric vessels (SIE) relative to abdominal wall (dotted line). There is no superficial inferior artery in the immune-compromised hooded pigmented Hsd:RH-$Foxn1^{rnu}$ strain (ii).

Figure EV5. Combination therapy with TNF/Mel/SM and checkpoint inhibitors further prolongs recurrence-free survival following ILP.

A Flow cytometry assay demonstrating the cross-reactivity of the 9H10 CTLA-4 clone to the rat CTLA-4 protein.

B–G Individual growth curves comparing controls (B) with animals treated with ILP-anti-CTLA-4 (C), ILP-anti-PD-1 (D), ILP-TNF/Mel/SM alone (E) or ILP-TNF/Mel/SM in combination with anti-CTLA-4 (F) or anti-PD-1 (G) (*n* = 6 per cohort).

H–J Tumours were collected 4 days after the ILP procedure and analysed by flow cytometry (*n* = 3 per cohort). No significant alteration was noted in $CD3^+CD161^-$ T cells (H), $CD3^+CD8^+$ T cells (I) or $CD4^+$ T-cell ICOS expression (J) (*n* = 3 per cohort). SM represents Birinapant. Error bars represent SD. Abbreviations: positive (+ve), negative (−ve).

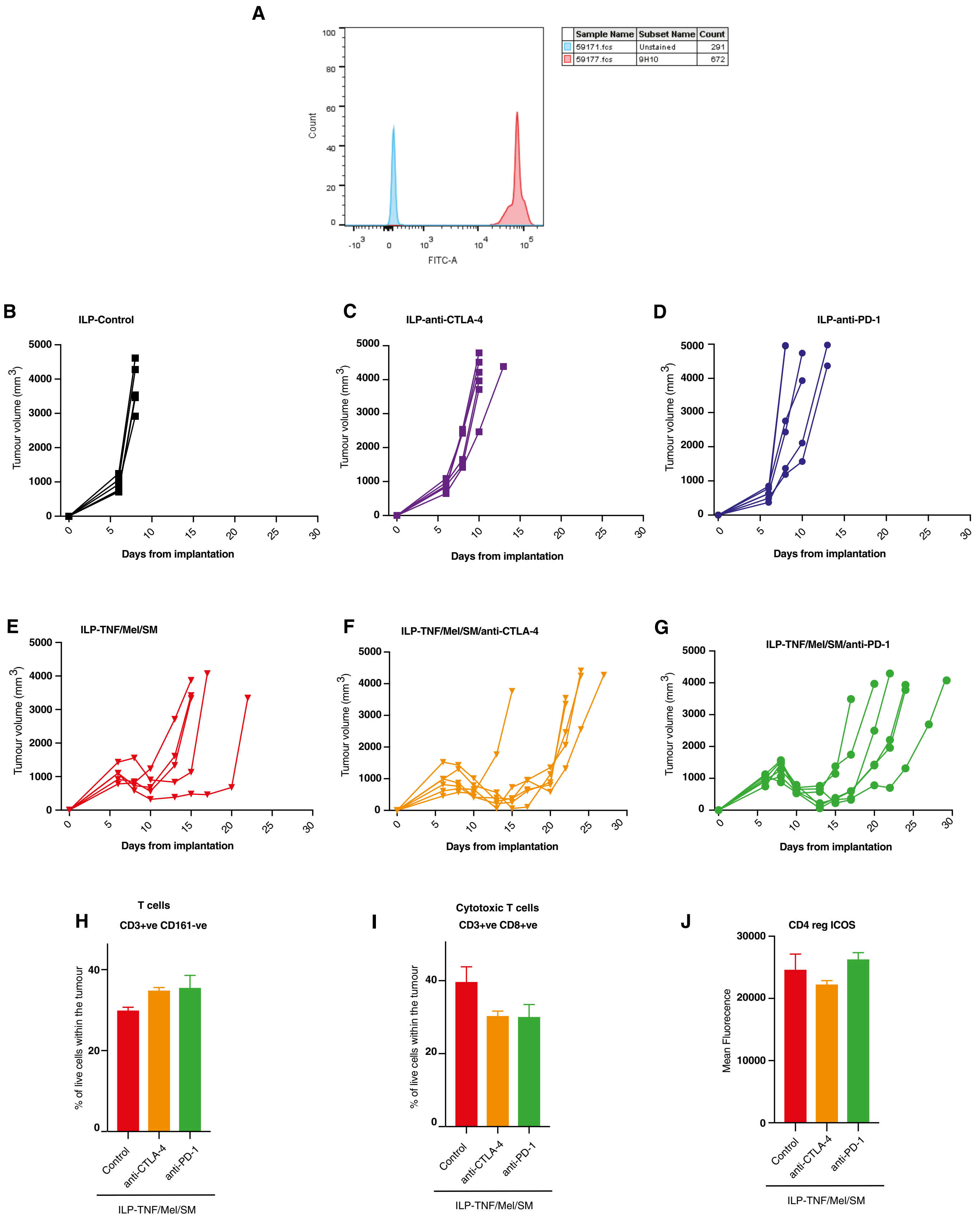

Figure EV5.

 © 2020 The Authors

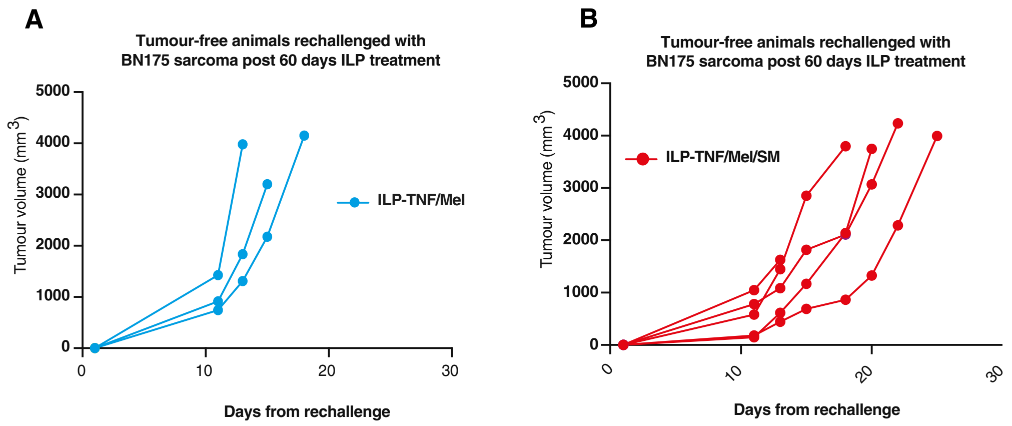

Figure EV6. SM enhances anti-tumour immunity.

A, B Individual tumour growth curves on re-challenge with BN175 sarcoma on contralateral limb of long-term survivors ILP-TNF/Mel (A) (n = 3 per cohort) or ILP-TNF/Mel/SM (B) (n = 5 per cohort). SM represents Birinapant.
