# Supplementary figures and images for: RIPK1‐mediated immunogenic cell death promotes anti‐tumour immunity against soft‐tissue sarcoma
Source: EMBO Mol Med. 2020 May 18;12(6):e10979. doi: 10.15252/emmm.201910979 (PMC7278545; doi:10.15252/emmm.201910979)

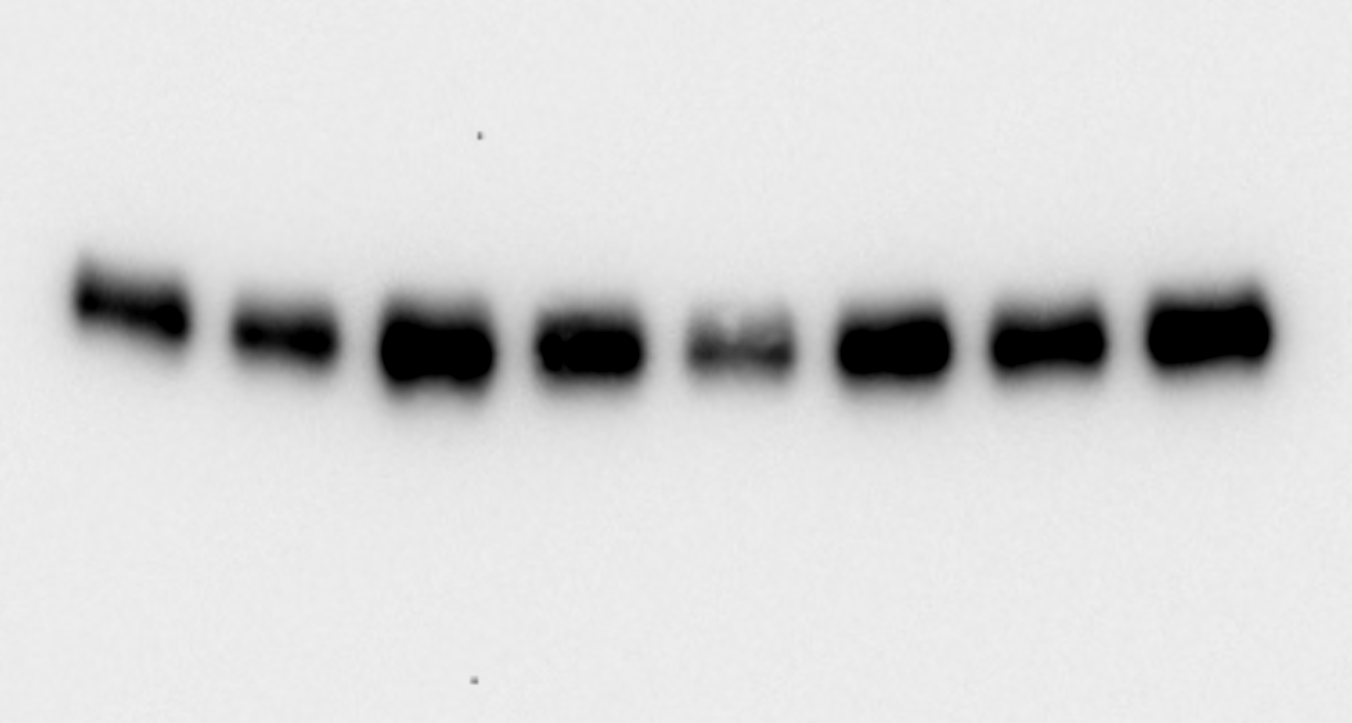

Supplement: Supplementary file 4 — Source Data for Figure 1 [file EMMM-12-e10979-s003.zip › Figure 1/Figure 1B/FADD.tif]

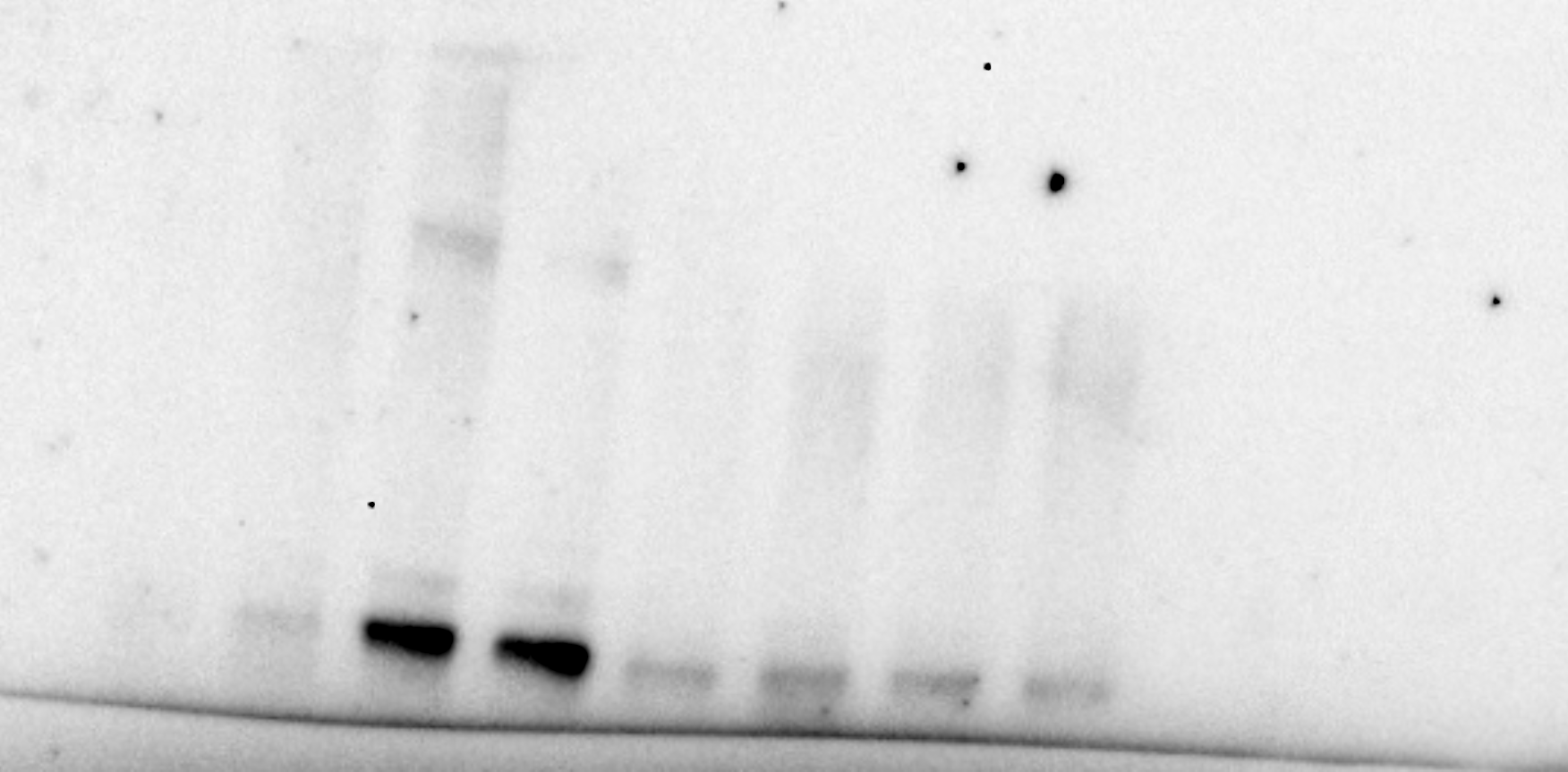

Supplement: Supplementary file 4 — Source Data for Figure 1 [file EMMM-12-e10979-s003.zip › Figure 1/Figure 1B/RIPK1 IP.tif]

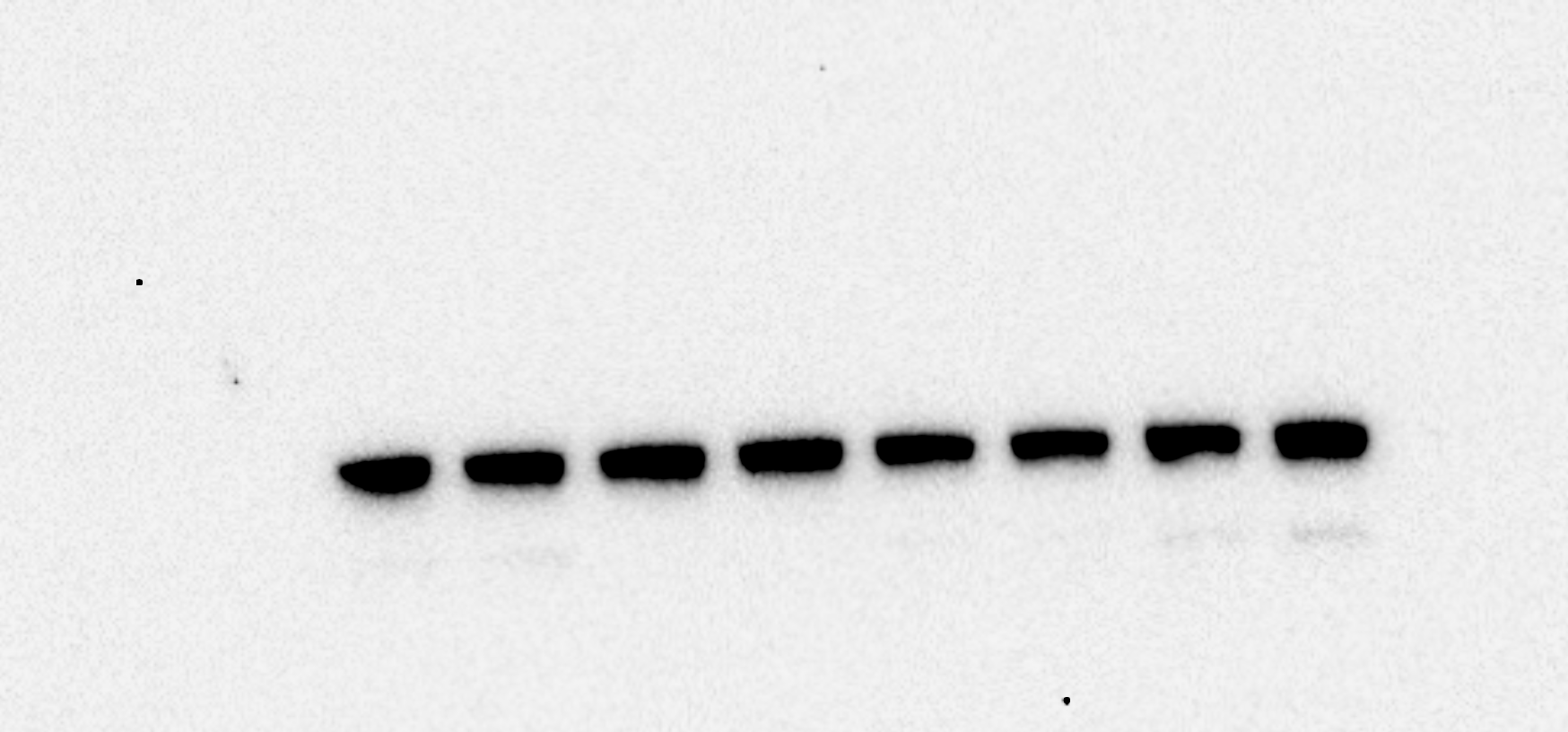

Supplement: Supplementary file 4 — Source Data for Figure 1 [file EMMM-12-e10979-s003.zip › Figure 1/Figure 1B/hsp90.tif]

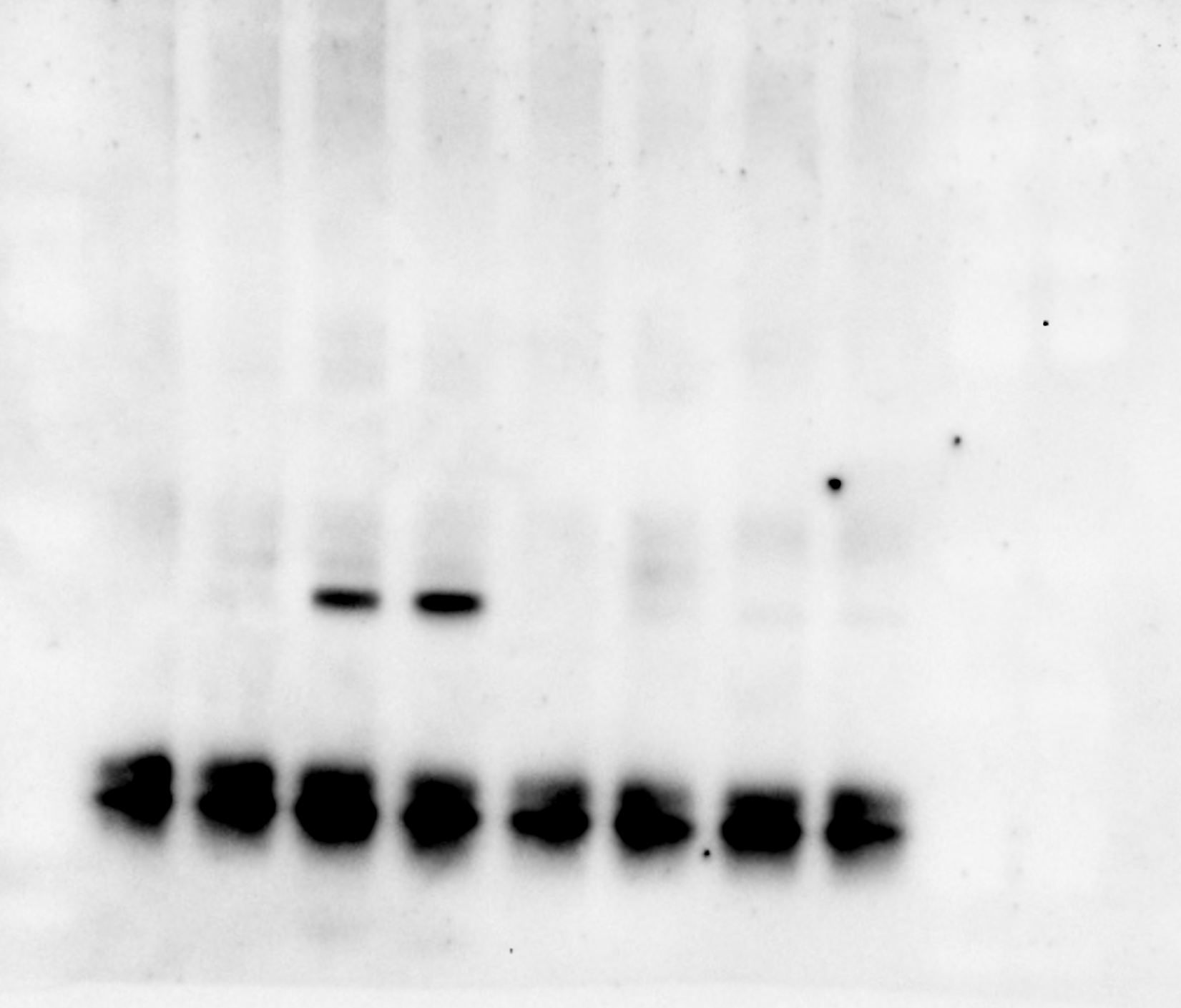

Supplement: Supplementary file 4 — Source Data for Figure 1 [file EMMM-12-e10979-s003.zip › Figure 1/Figure 1B/C8 IP.tif]

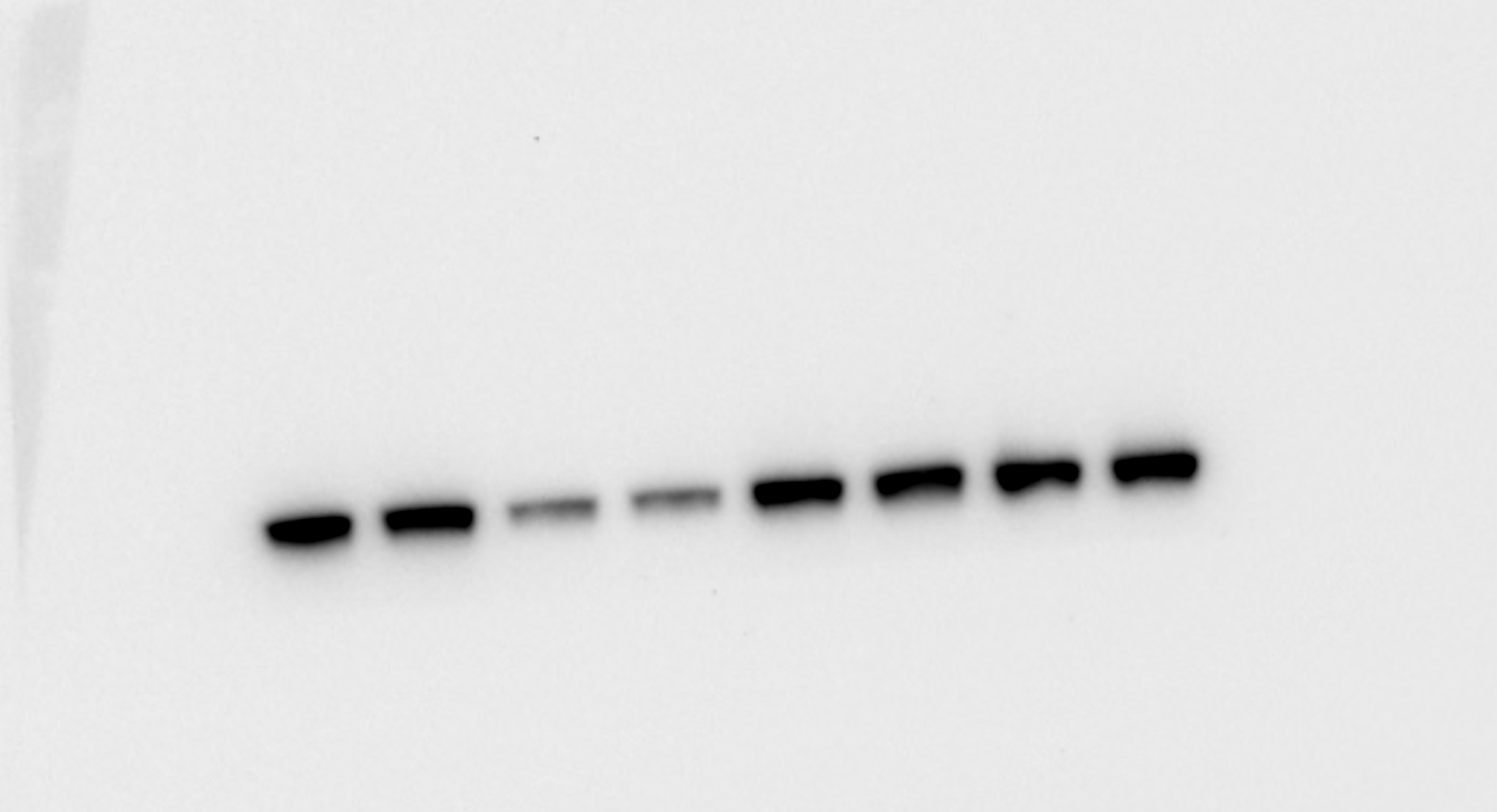

Supplement: Supplementary file 4 — Source Data for Figure 1 [file EMMM-12-e10979-s003.zip › Figure 1/Figure 1B/RIPK1 IN.tif]

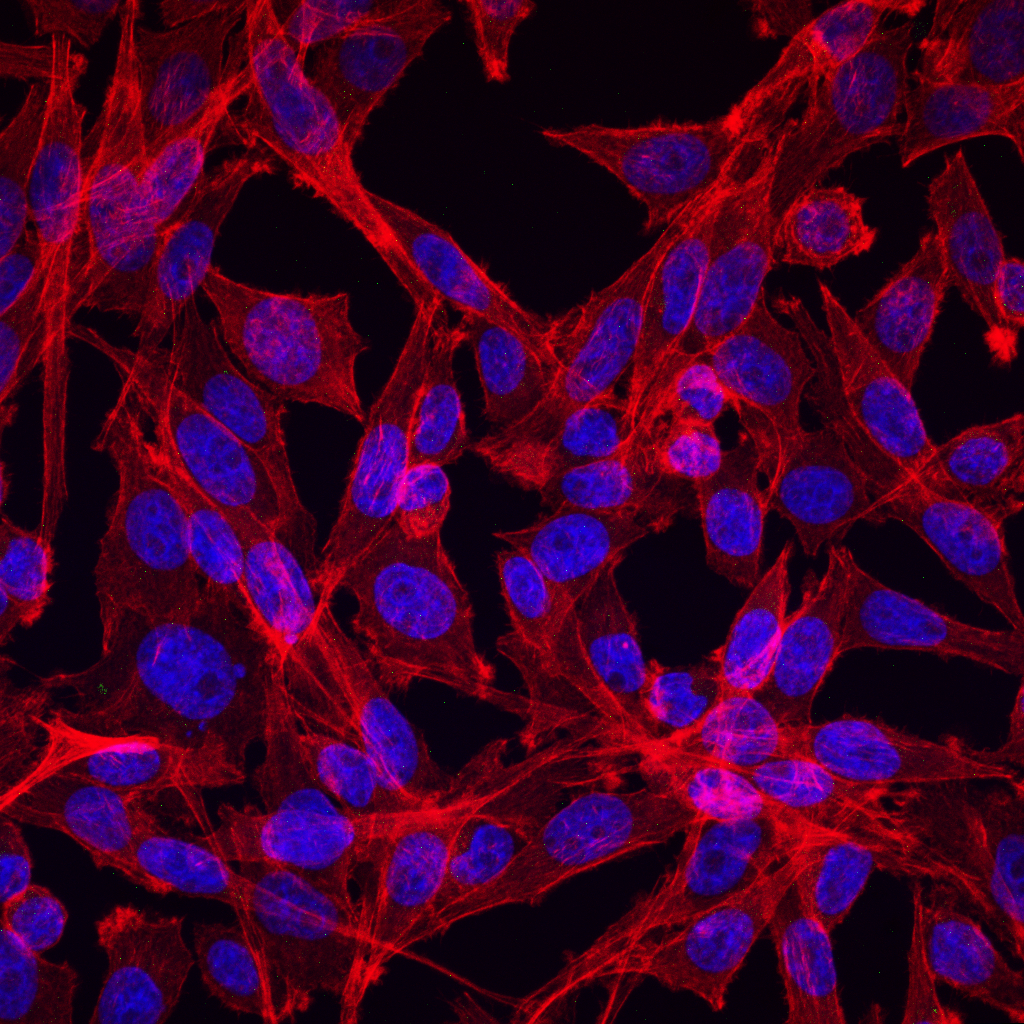

Supplement: Supplementary file 4 — Source Data for Figure 1 [file EMMM-12-e10979-s003.zip › Figure 1/Figure 1C/SM/SM_Maximumintensityprojection2.tif (RGB).tif]

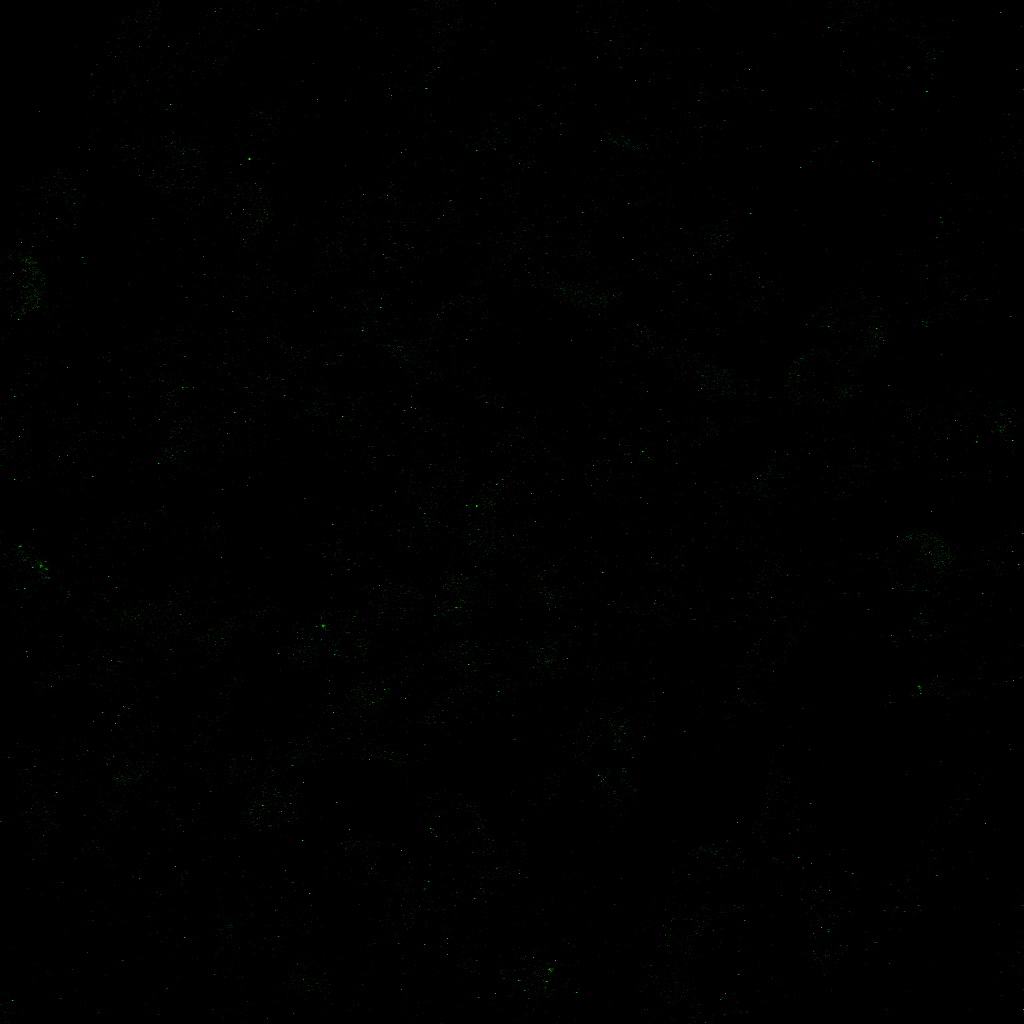

Supplement: Supplementary file 4 — Source Data for Figure 1 [file EMMM-12-e10979-s003.zip › Figure 1/Figure 1C/Mel:SM/MELSM_green2.tif (RGB).tif]

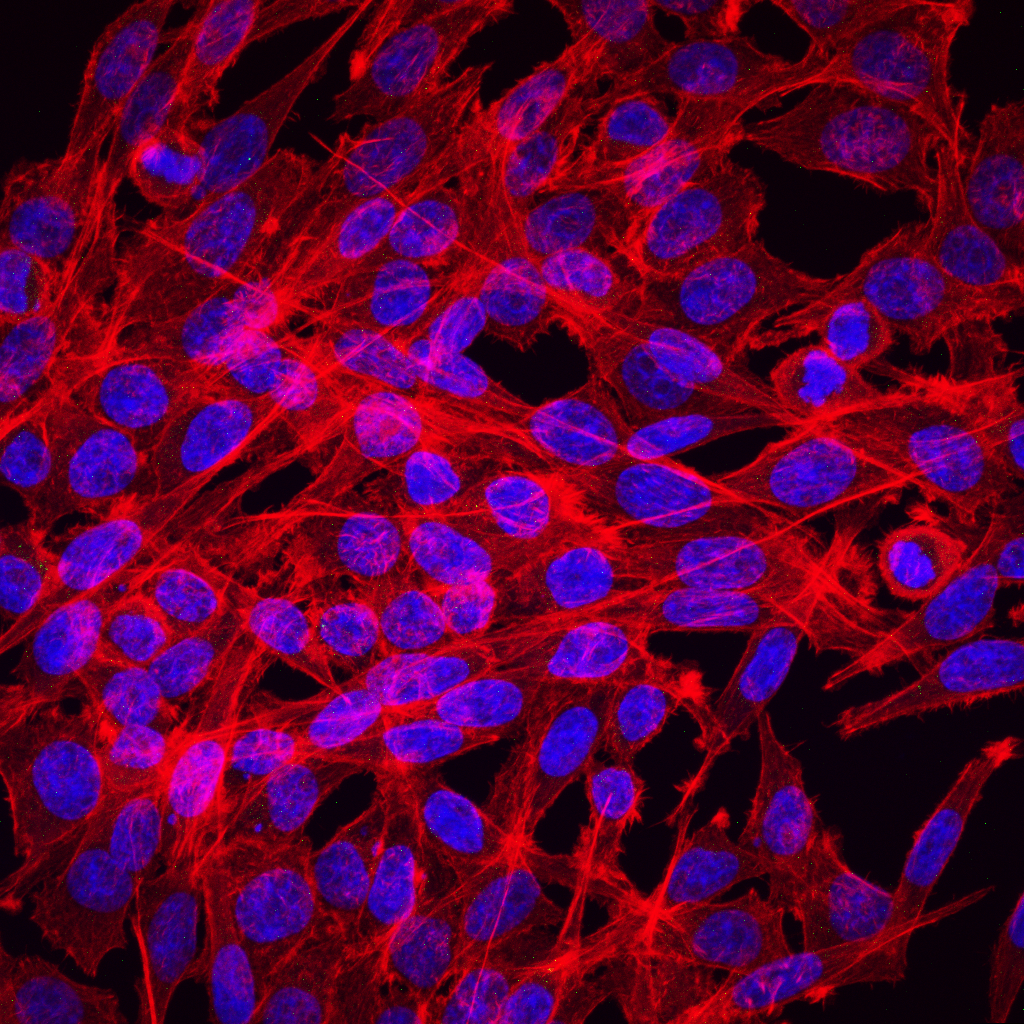

Supplement: Supplementary file 4 — Source Data for Figure 1 [file EMMM-12-e10979-s003.zip › Figure 1/Figure 1C/Mel:SM/MELSM_Maximumintensityprojection2.tif (RGB).tif]

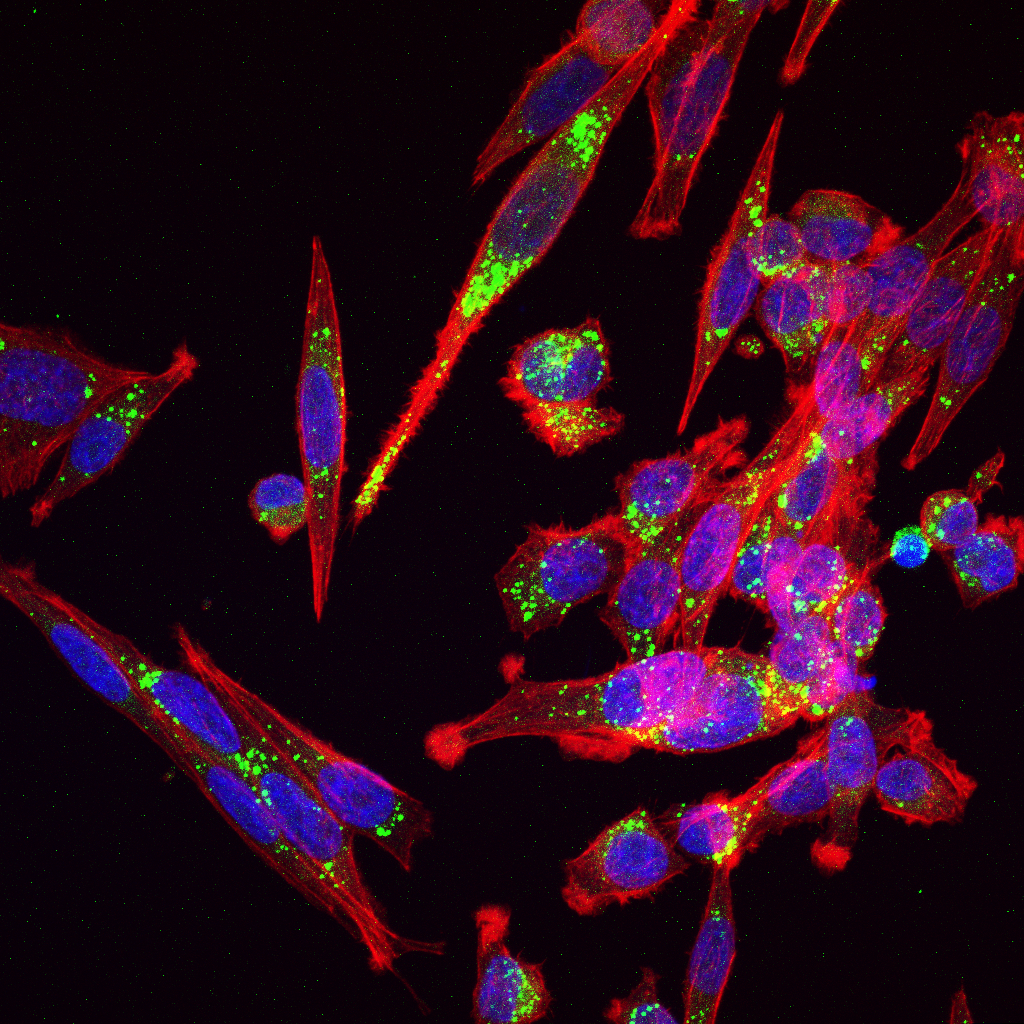

Supplement: Supplementary file 4 — Source Data for Figure 1 [file EMMM-12-e10979-s003.zip › Figure 1/Figure 1C/TNF:Mel:SM/TNF_Mel_SM_Maximumintensityprojection.tif (RGB).png]

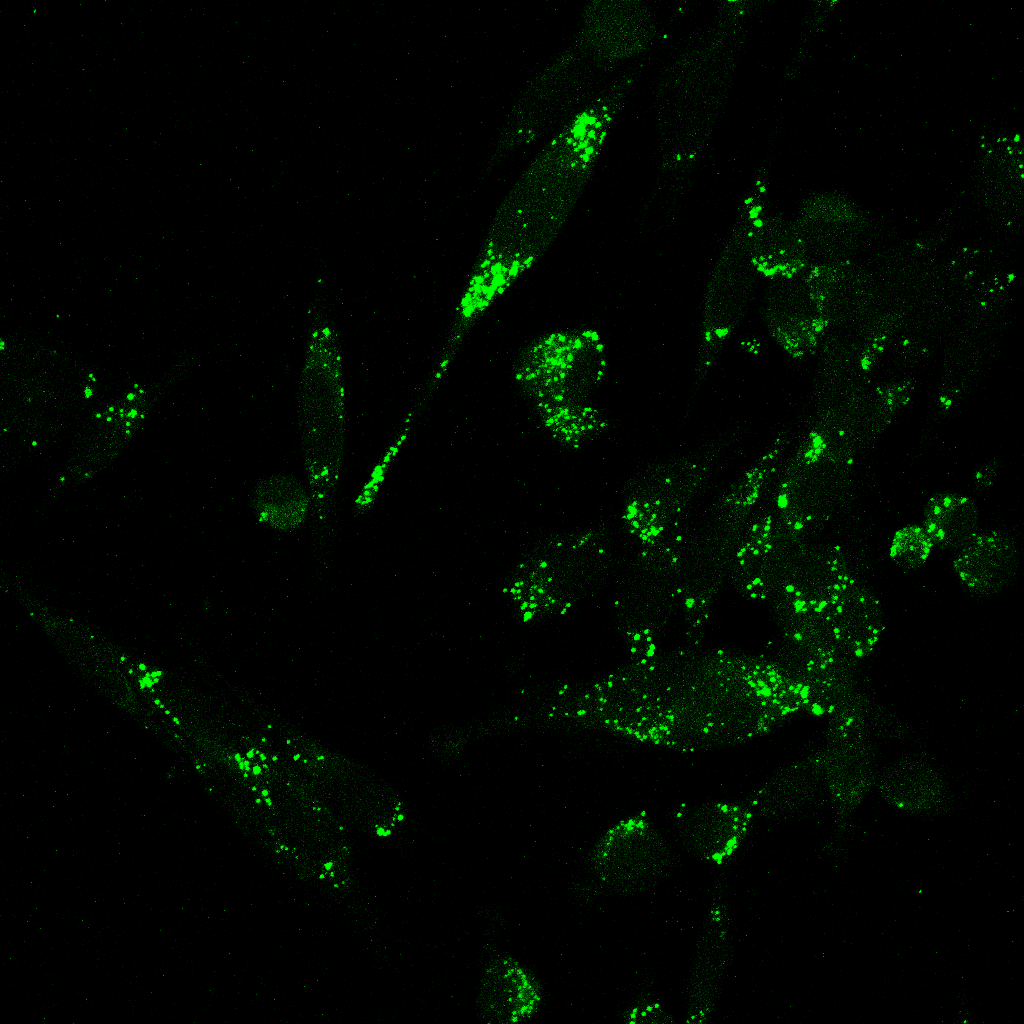

Supplement: Supplementary file 4 — Source Data for Figure 1 [file EMMM-12-e10979-s003.zip › Figure 1/Figure 1C/TNF:Mel:SM/TNFMELSM Green.png]

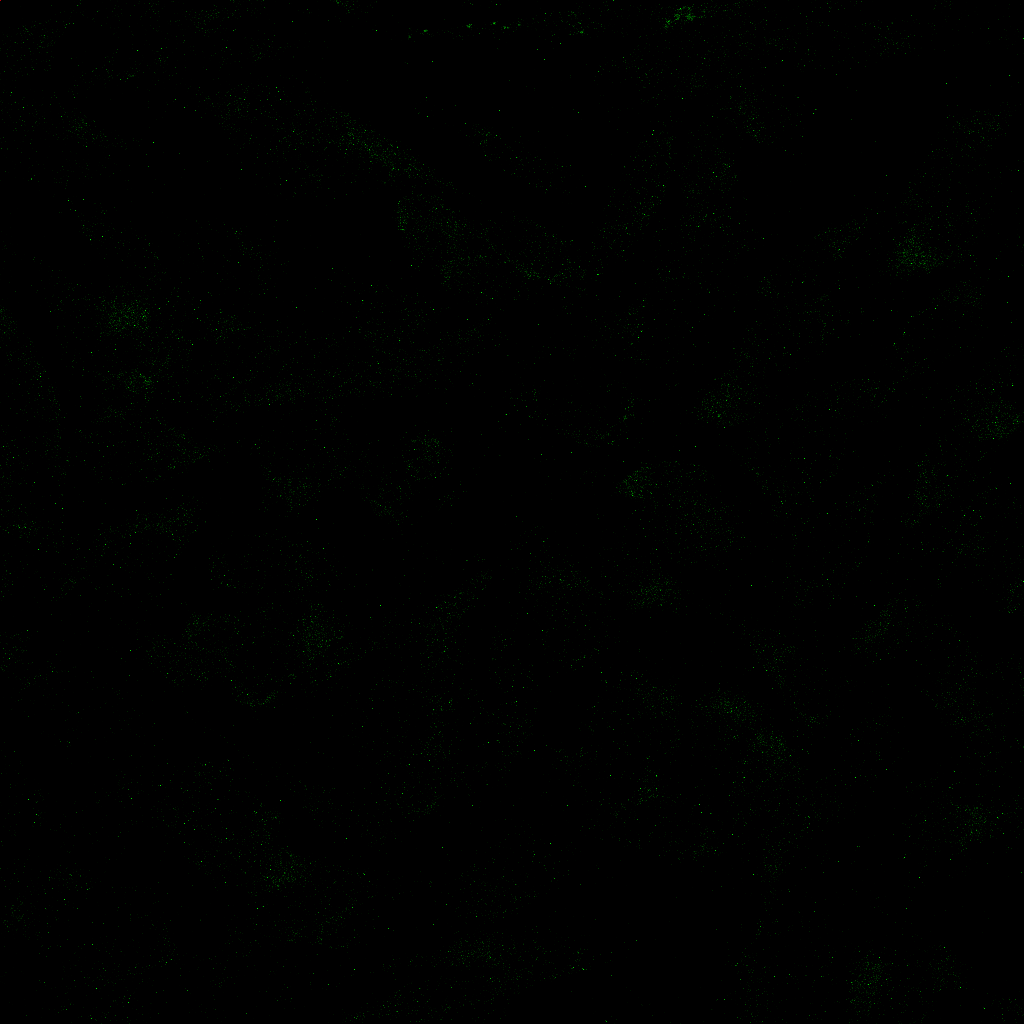

Supplement: Supplementary file 4 — Source Data for Figure 1 [file EMMM-12-e10979-s003.zip › Figure 1/Figure 1C/TNF/TNF_green2.tif (RGB).tif]

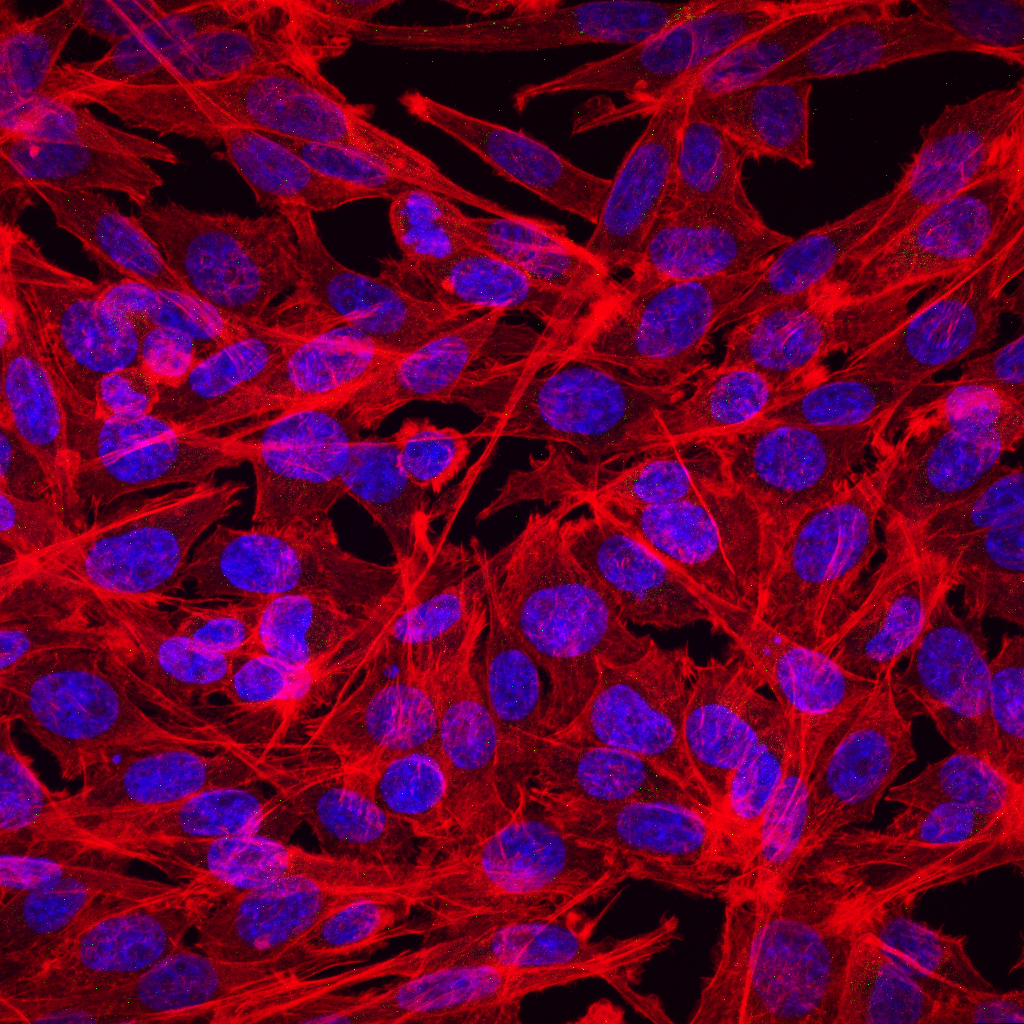

Supplement: Supplementary file 4 — Source Data for Figure 1 [file EMMM-12-e10979-s003.zip › Figure 1/Figure 1C/TNF/TNF_Maximumintensityprojection2.tif (RGB).tif]

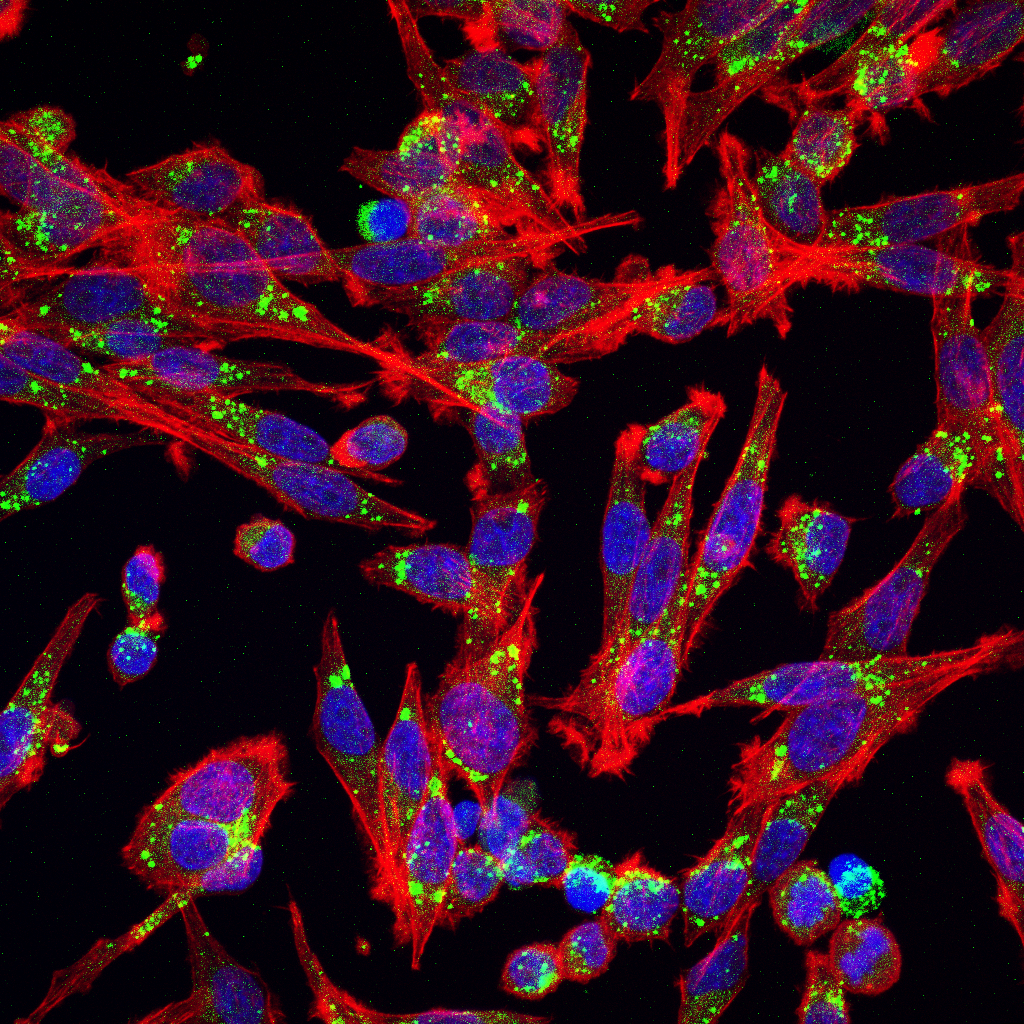

Supplement: Supplementary file 4 — Source Data for Figure 1 [file EMMM-12-e10979-s003.zip › Figure 1/Figure 1C/TNF:SM/TNFSM_Maximumintensityprojection2.tif (RGB).tif]

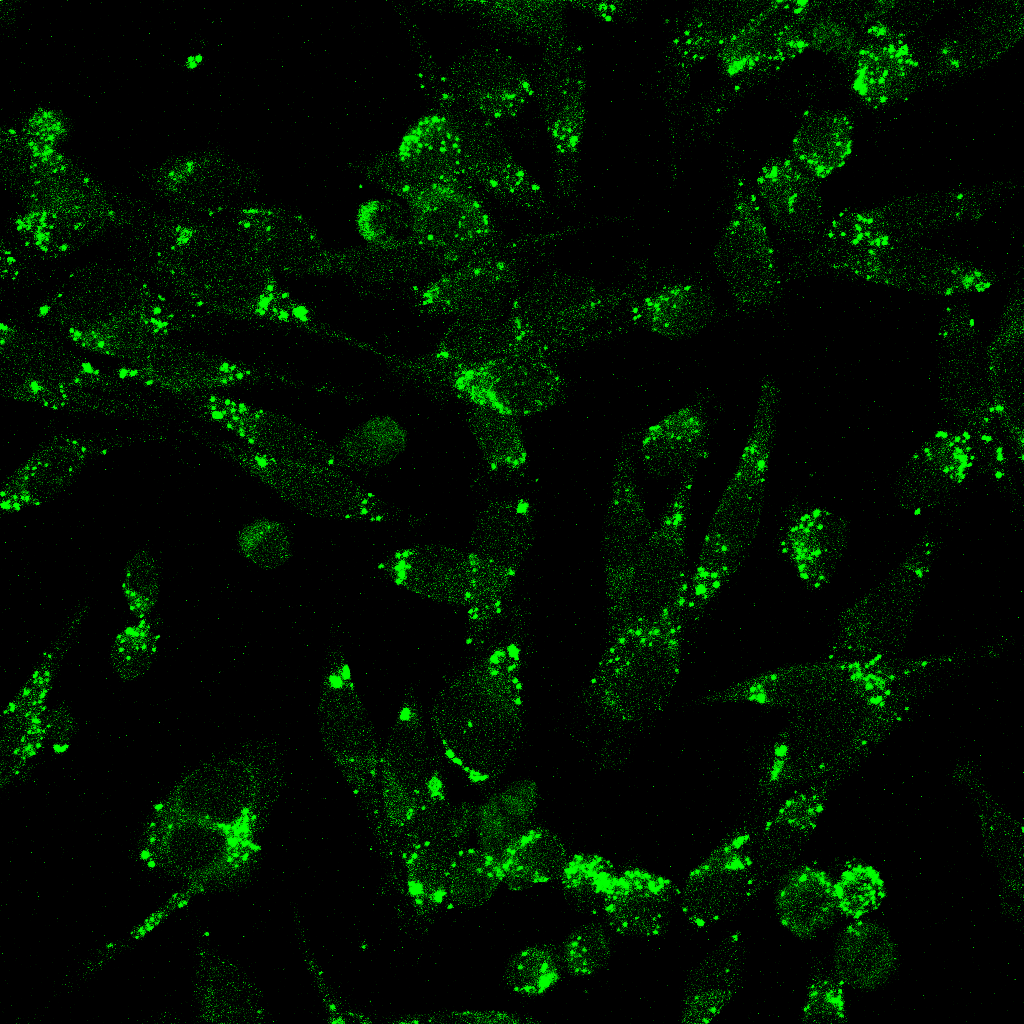

Supplement: Supplementary file 4 — Source Data for Figure 1 [file EMMM-12-e10979-s003.zip › Figure 1/Figure 1C/TNF:SM/TNFSM_green2.tif]

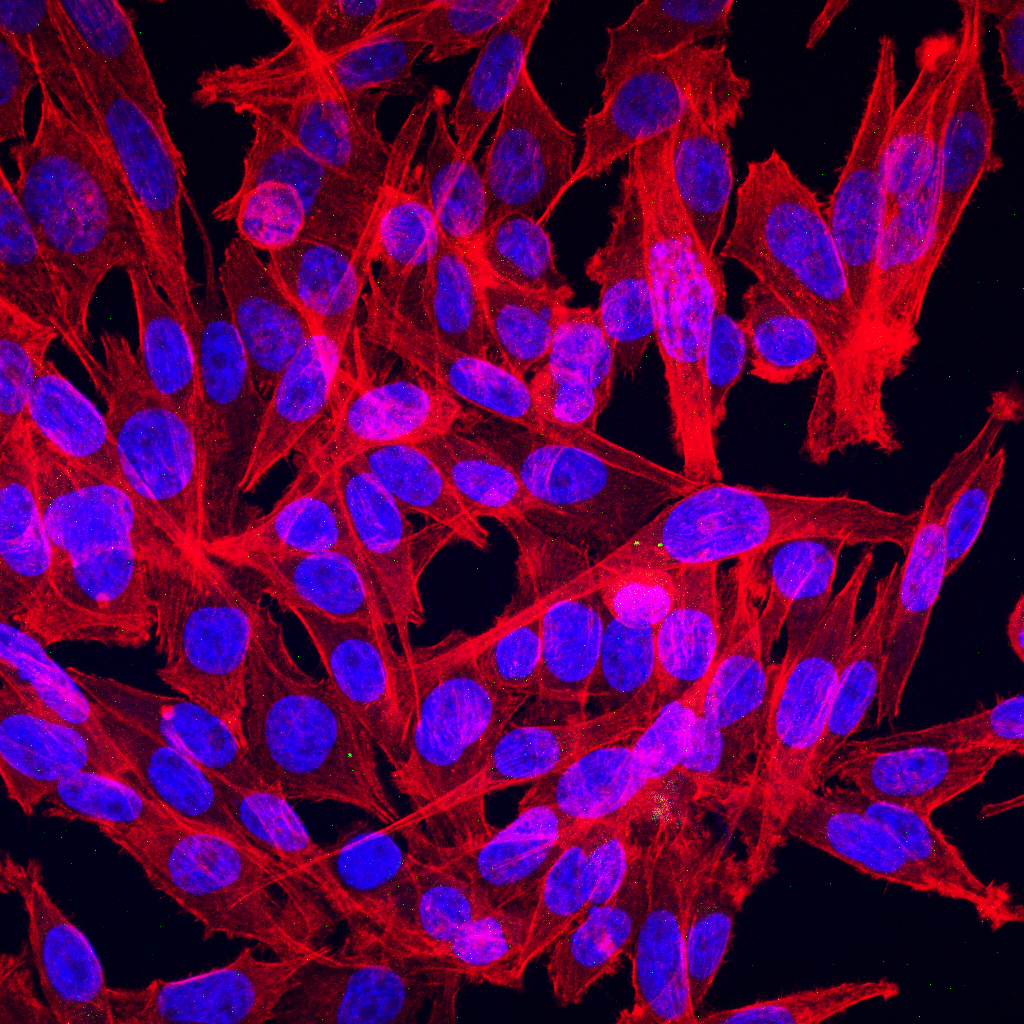

Supplement: Supplementary file 4 — Source Data for Figure 1 [file EMMM-12-e10979-s003.zip › Figure 1/Figure 1C/TNF:Mel/TNFMel_Maximumintensityprojection2.tif (RGB).tif]

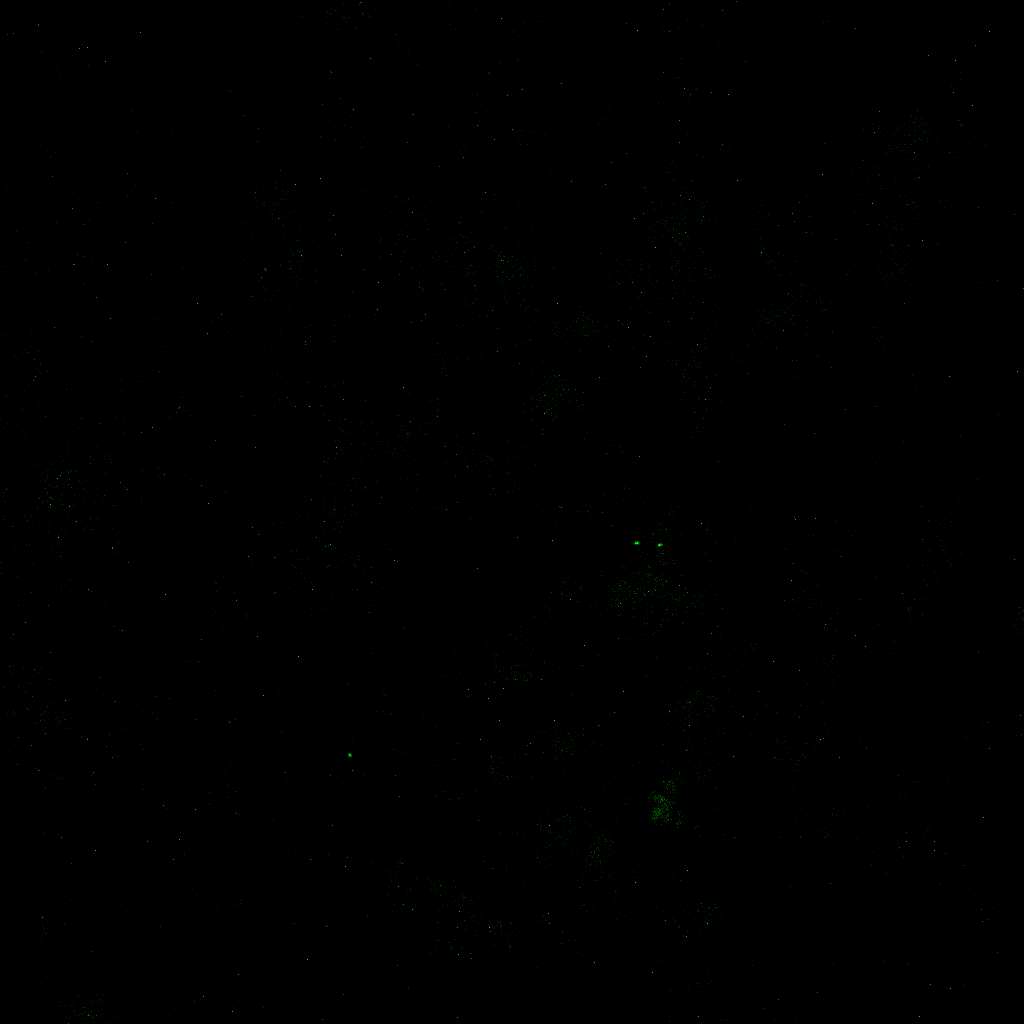

Supplement: Supplementary file 4 — Source Data for Figure 1 [file EMMM-12-e10979-s003.zip › Figure 1/Figure 1C/TNF:Mel/TNFMel_green2.tif (RGB).tif]

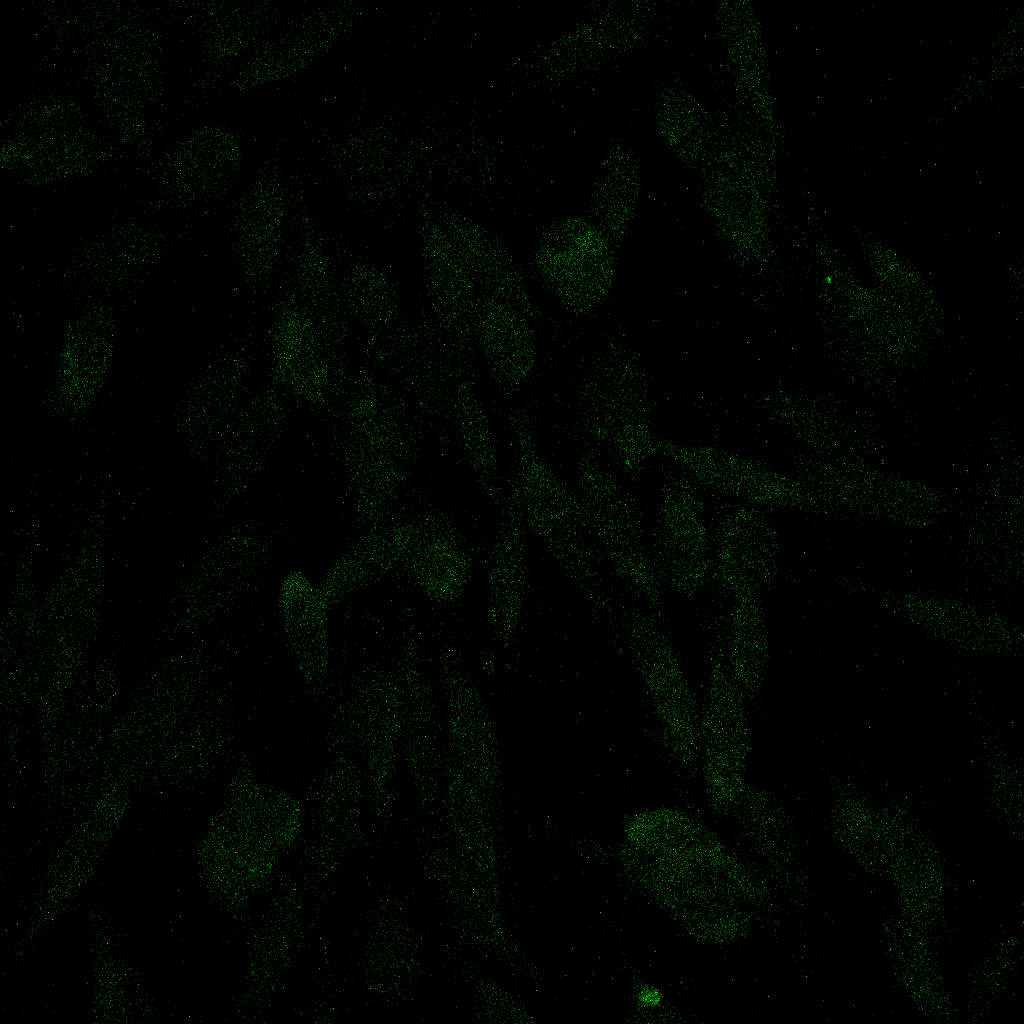

Supplement: Supplementary file 4 — Source Data for Figure 1 [file EMMM-12-e10979-s003.zip › Figure 1/Figure 1C/Unt/Unt_green2.tif (RGB).tif]

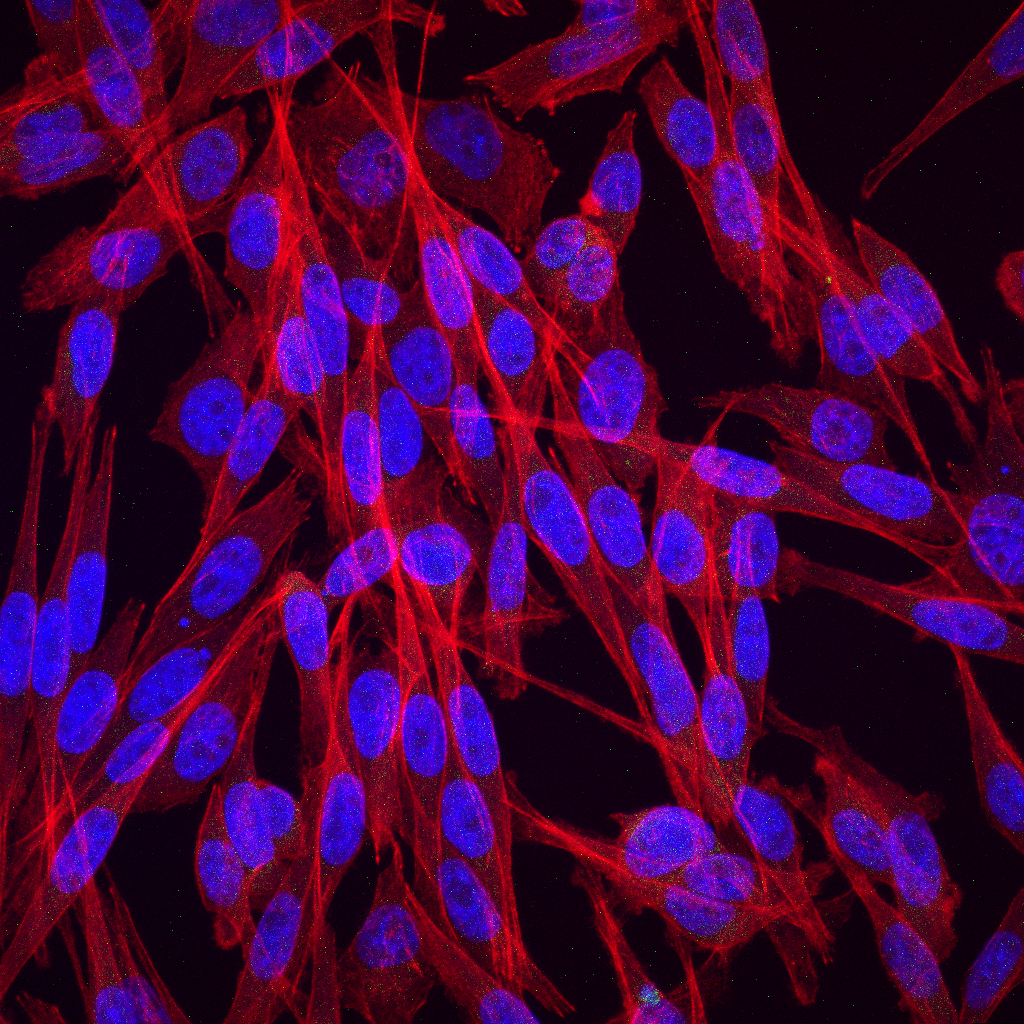

Supplement: Supplementary file 4 — Source Data for Figure 1 [file EMMM-12-e10979-s003.zip › Figure 1/Figure 1C/Unt/Unt_Maximumintensityprojection2.tif (RGB).tif]

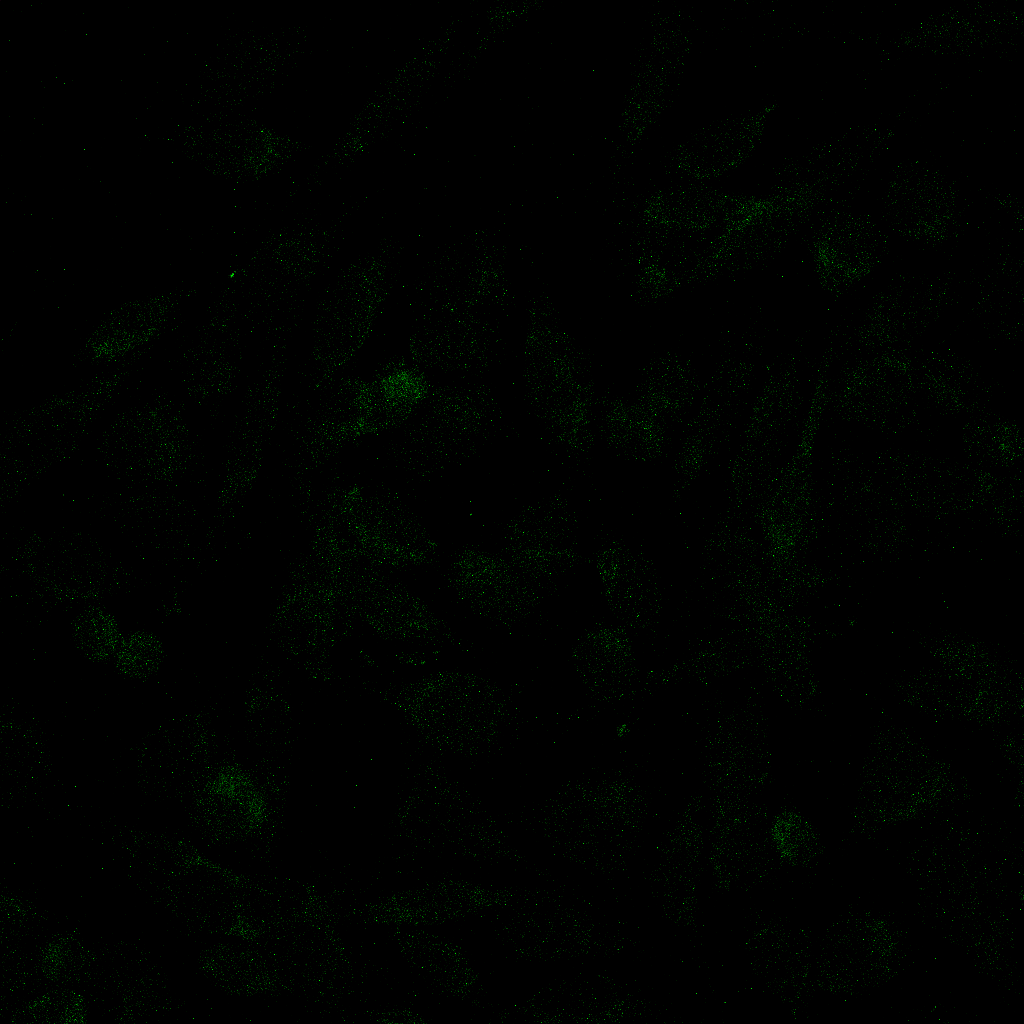

Supplement: Supplementary file 4 — Source Data for Figure 1 [file EMMM-12-e10979-s003.zip › Figure 1/Figure 1C/Mel/Mel_green.tif (RGB)2.tif]

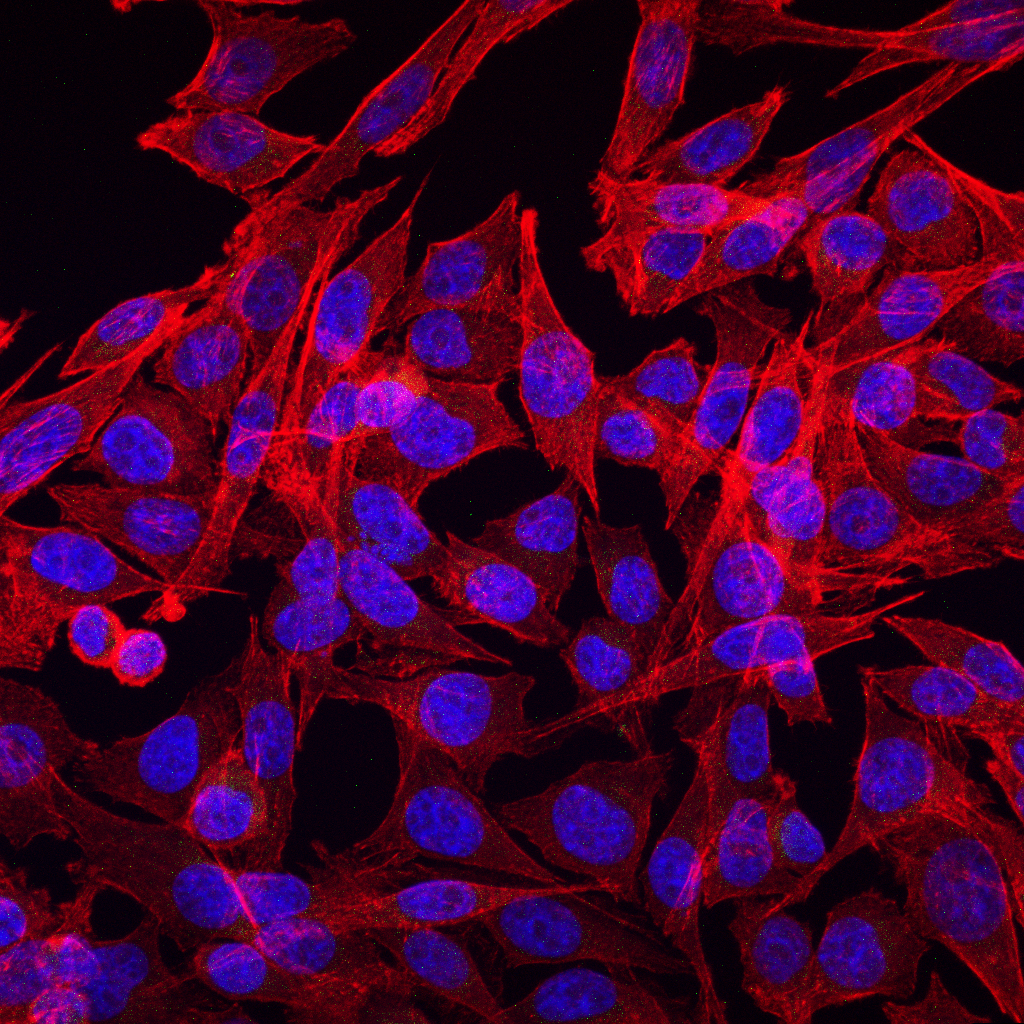

Supplement: Supplementary file 4 — Source Data for Figure 1 [file EMMM-12-e10979-s003.zip › Figure 1/Figure 1C/Mel/Mel_Maximumintensityprojection2.tif (RGB).tif]

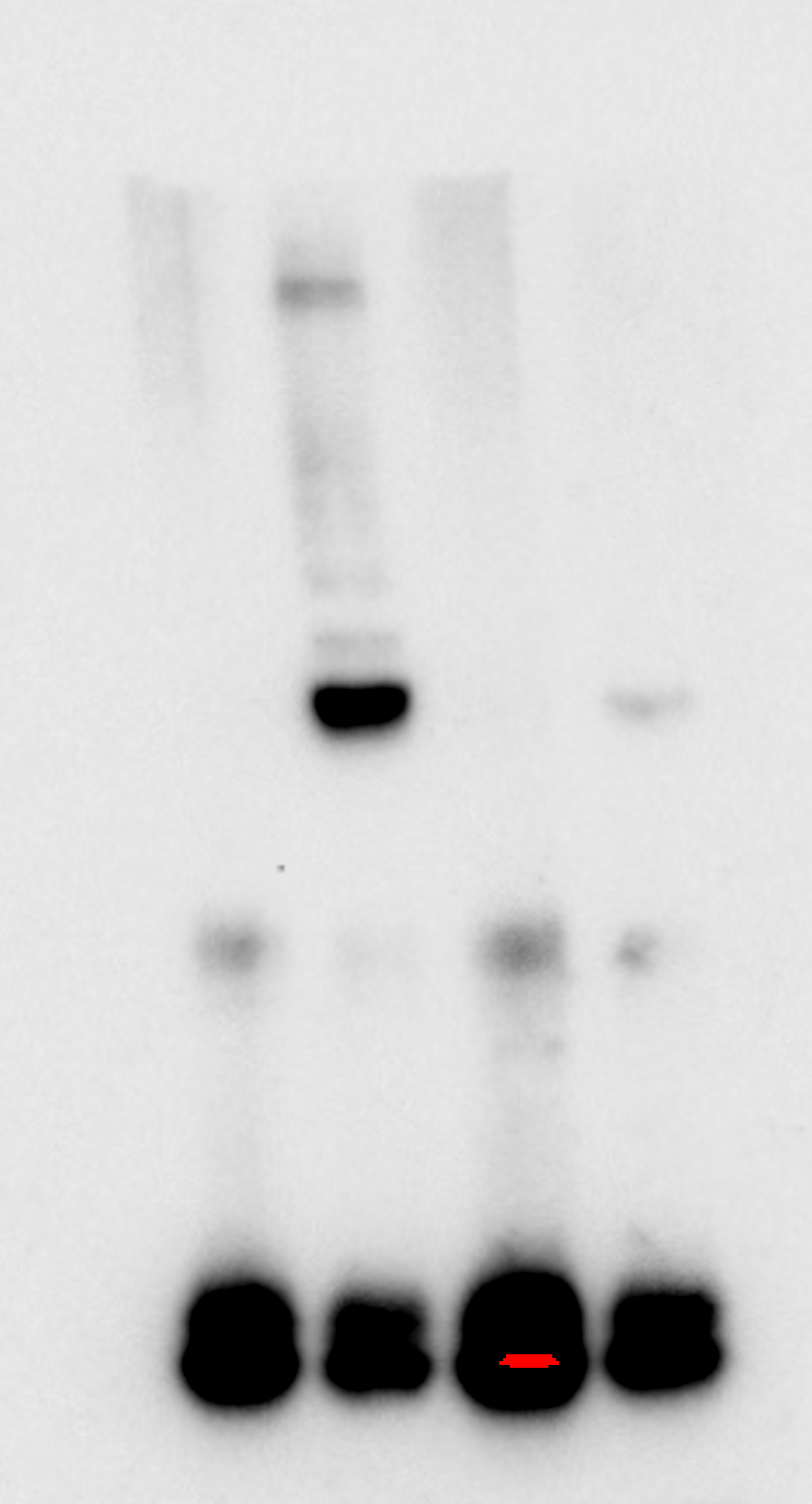

Supplement: Supplementary file 5 — Source Data for Figure 2 [file EMMM-12-e10979-s004.zip › Figure 2/Figure 2A/RIPK1 ip ht1080.tif]

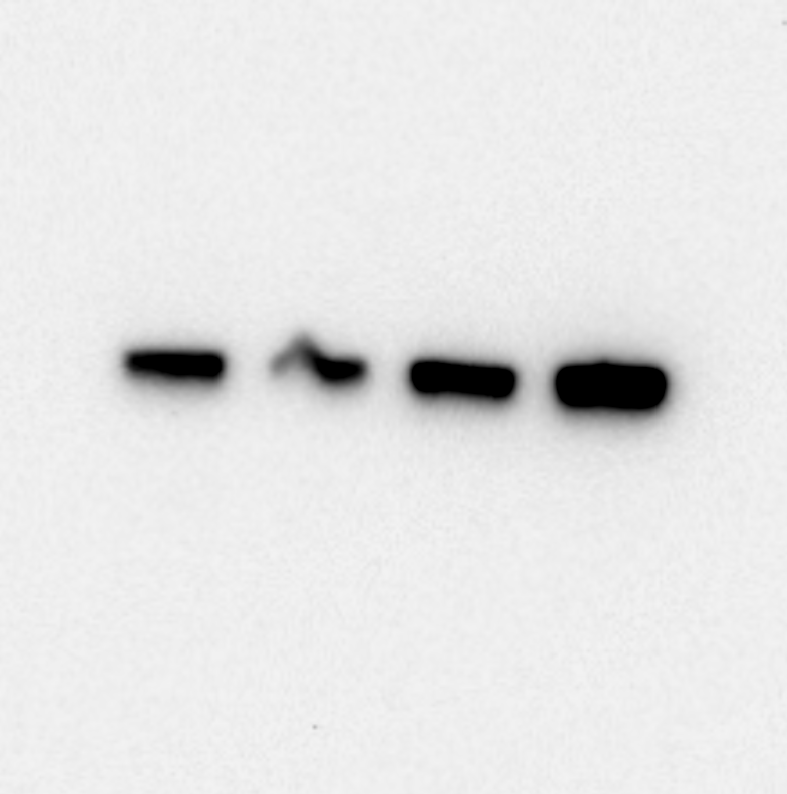

Supplement: Supplementary file 5 — Source Data for Figure 2 [file EMMM-12-e10979-s004.zip › Figure 2/Figure 2A/ripk1 in ht1080.tif]

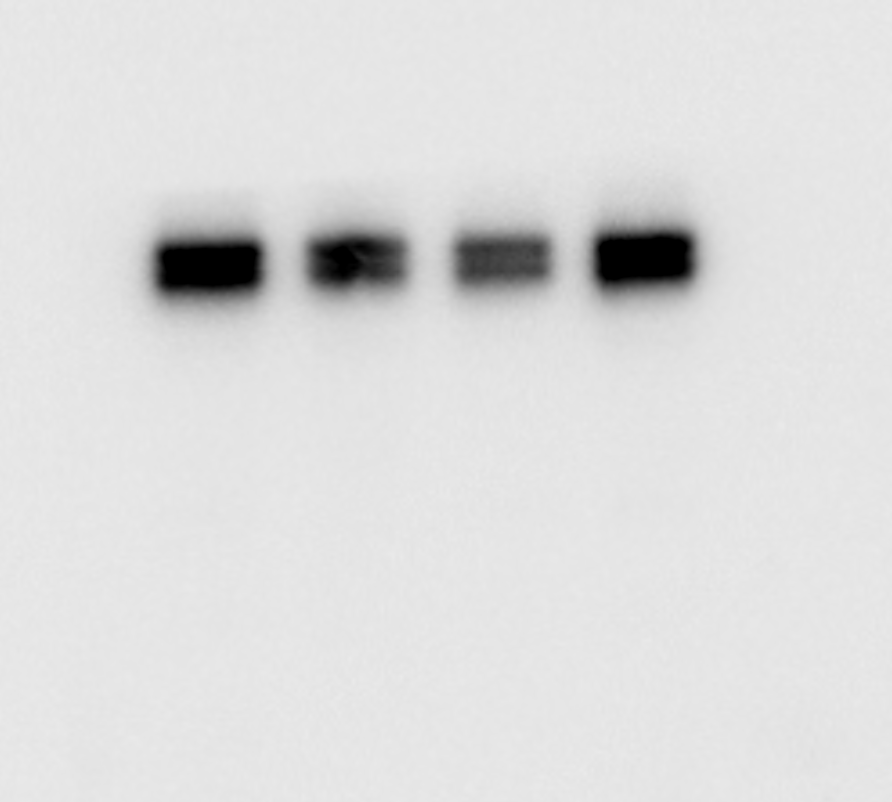

Supplement: Supplementary file 5 — Source Data for Figure 2 [file EMMM-12-e10979-s004.zip › Figure 2/Figure 2A/c8 IN.tif]

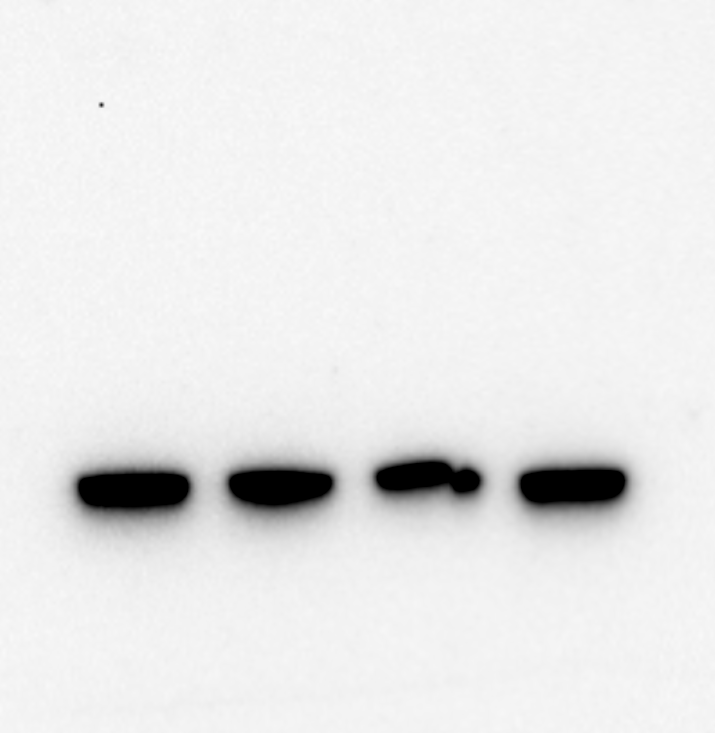

Supplement: Supplementary file 5 — Source Data for Figure 2 [file EMMM-12-e10979-s004.zip › Figure 2/Figure 2A/hsp90.tif]

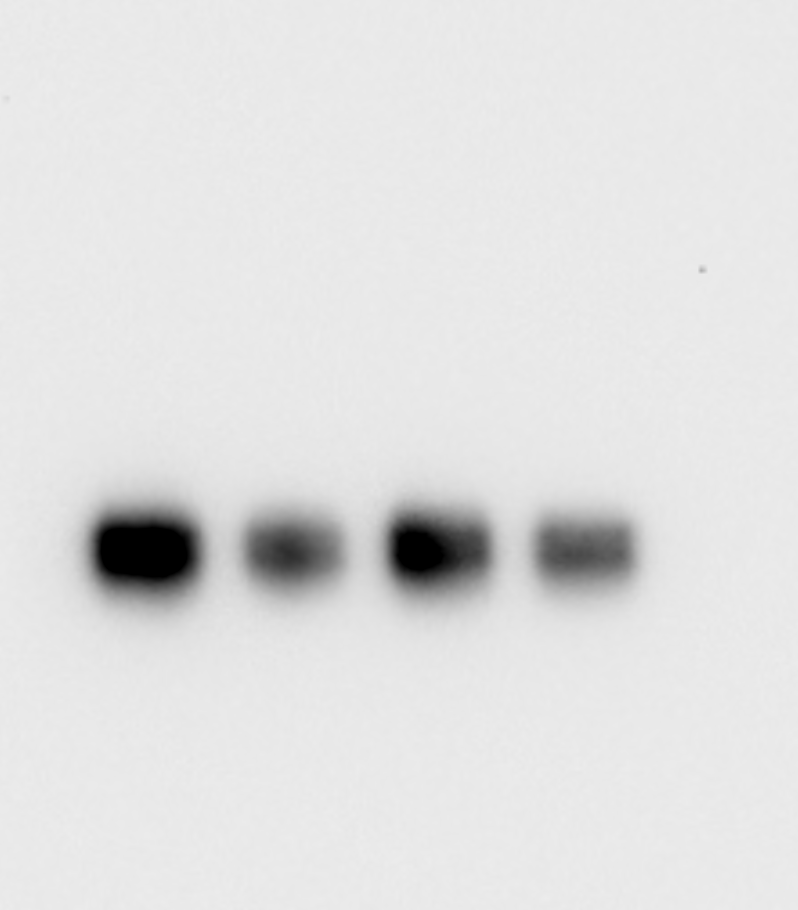

Supplement: Supplementary file 5 — Source Data for Figure 2 [file EMMM-12-e10979-s004.zip › Figure 2/Figure 2A/c8 IP.tif]

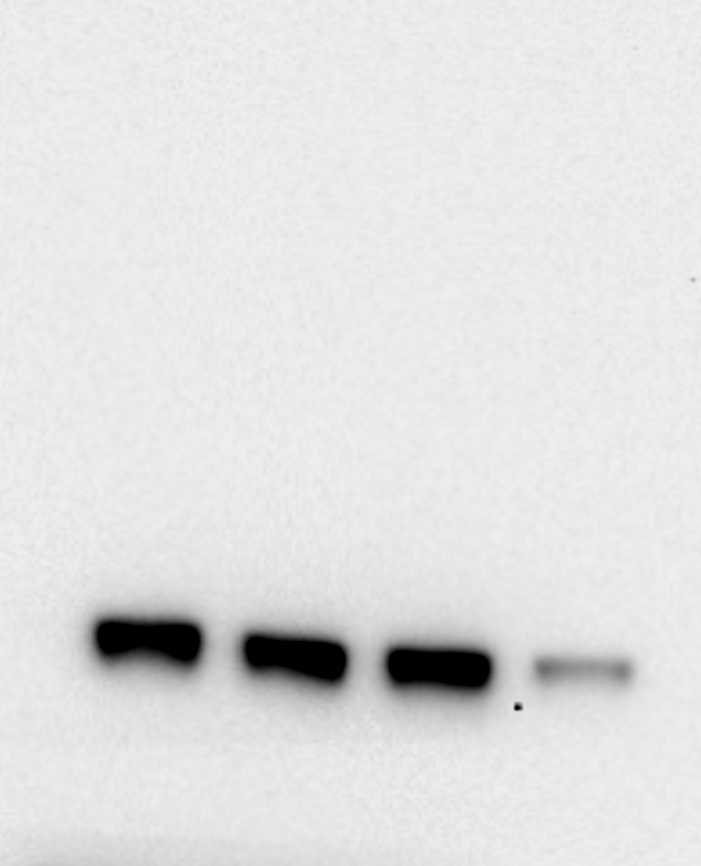

Supplement: Supplementary file 5 — Source Data for Figure 2 [file EMMM-12-e10979-s004.zip › Figure 2/Figure 2D/ripk1 in a375.tif]

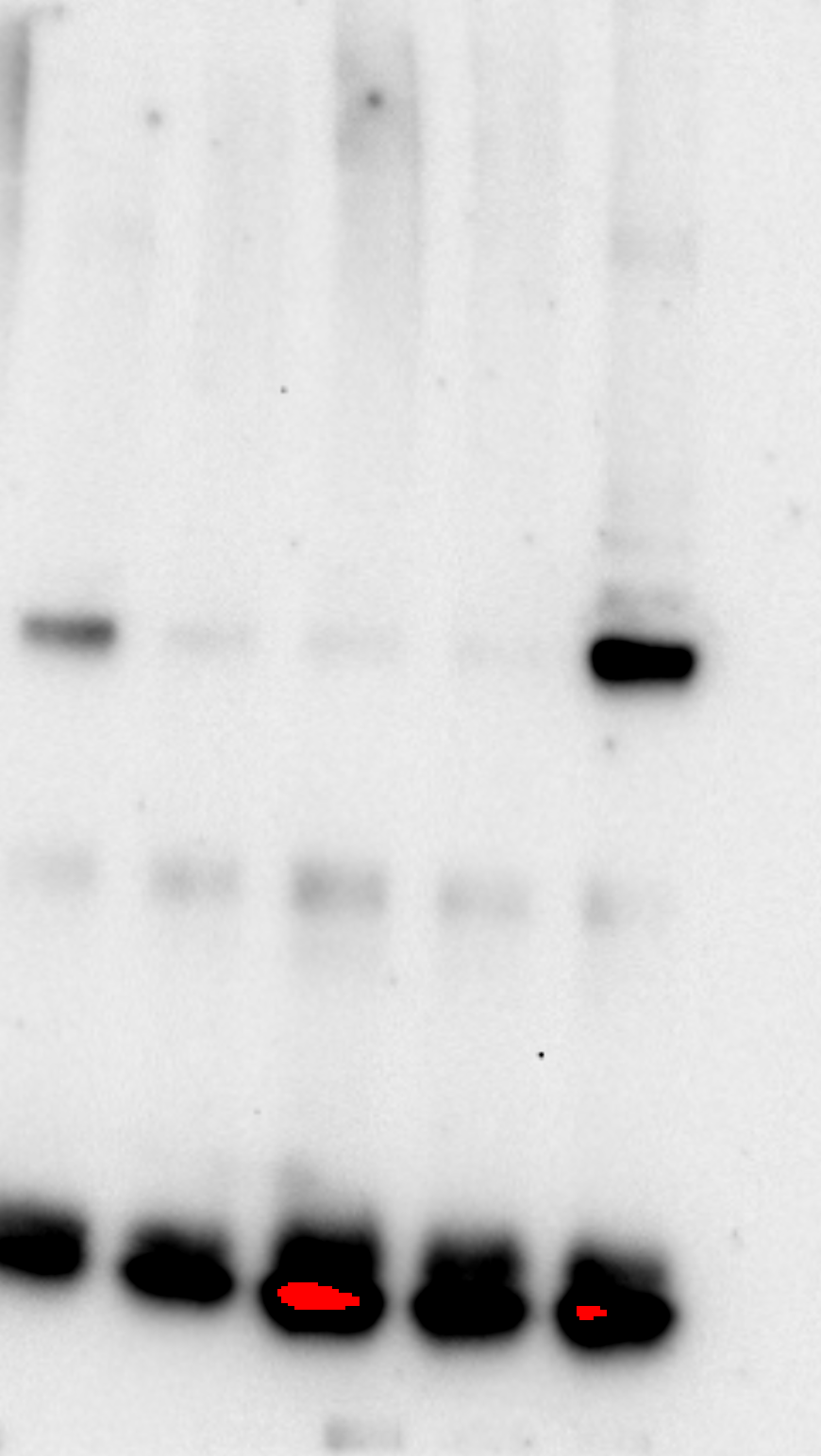

Supplement: Supplementary file 5 — Source Data for Figure 2 [file EMMM-12-e10979-s004.zip › Figure 2/Figure 2D/ripk1 IP.tif]

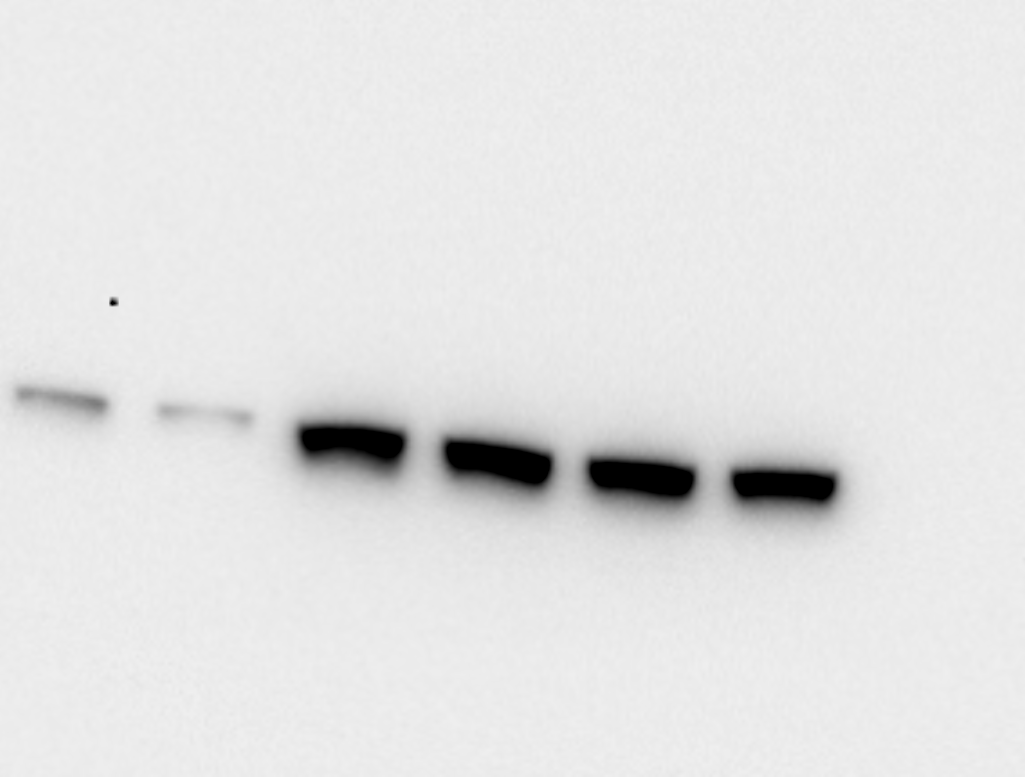

Supplement: Supplementary file 5 — Source Data for Figure 2 [file EMMM-12-e10979-s004.zip › Figure 2/Figure 2D/hsp90.tif]

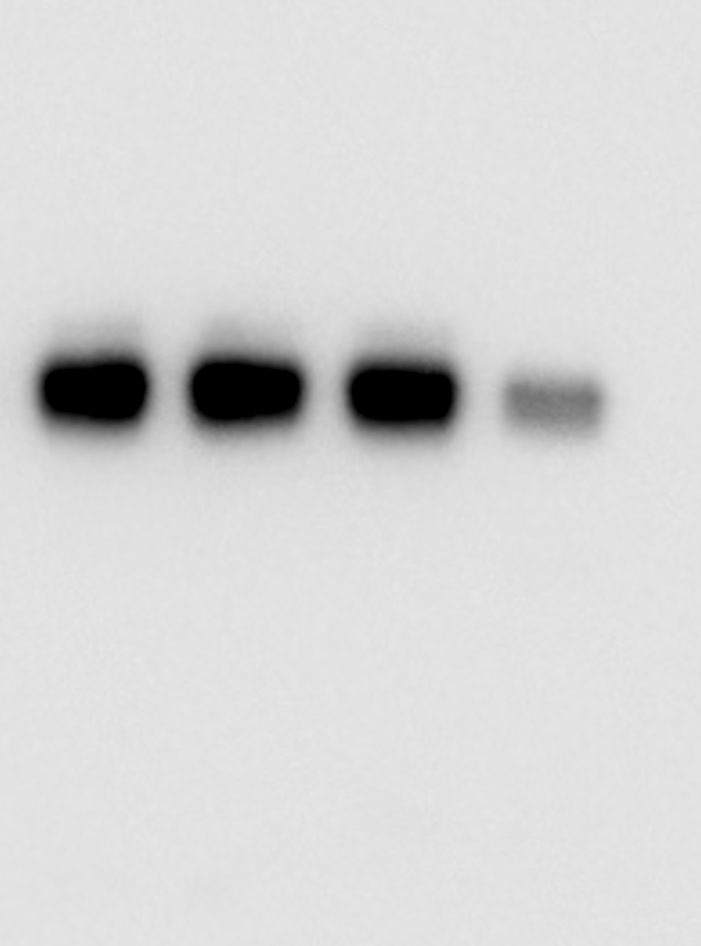

Supplement: Supplementary file 5 — Source Data for Figure 2 [file EMMM-12-e10979-s004.zip › Figure 2/Figure 2D/c8 IP.tif]

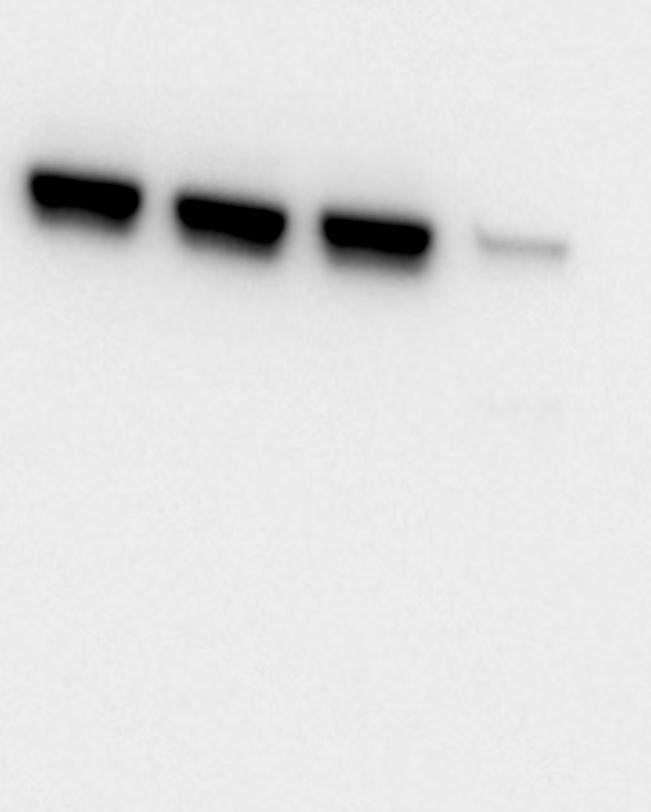

Supplement: Supplementary file 5 — Source Data for Figure 2 [file EMMM-12-e10979-s004.zip › Figure 2/Figure 2D/c8 in a375.tif]

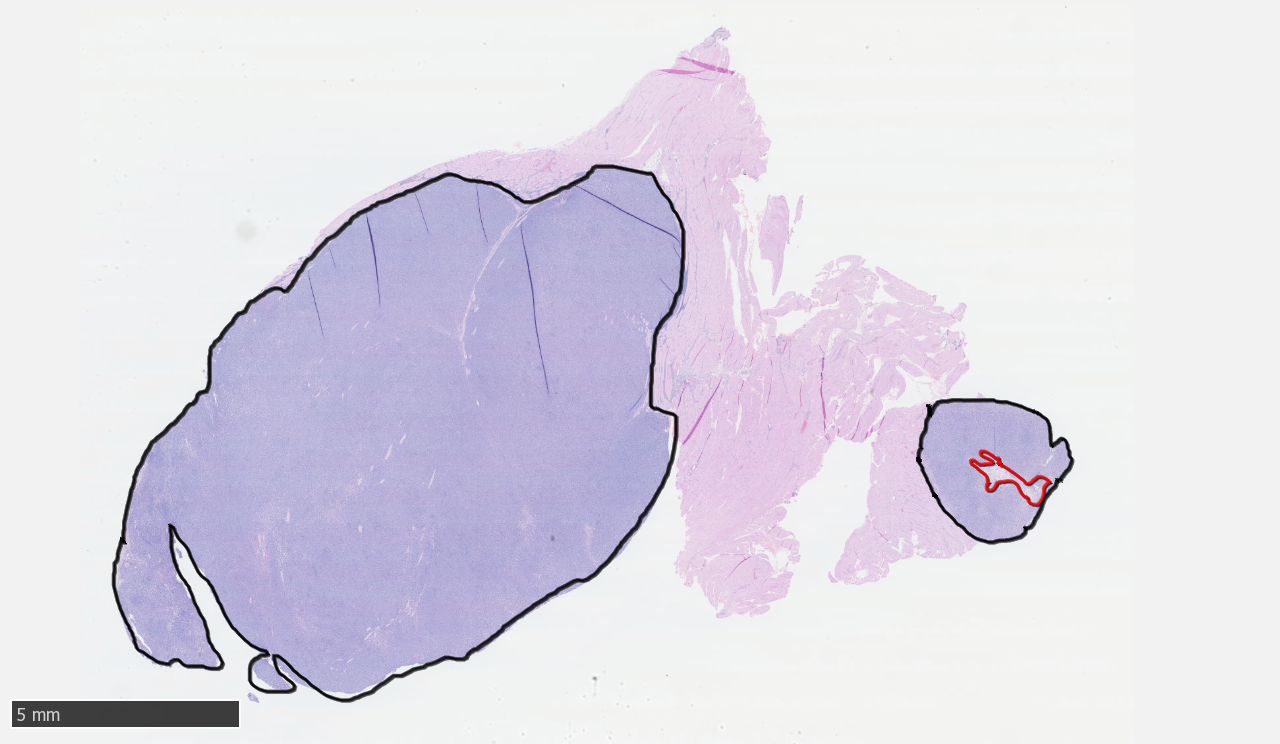

Supplement: Supplementary file 6 — Source Data for Figure 3 [file EMMM-12-e10979-s005.zip › Figure 3/Figure 3C ILP-TNF:Mel.tif]

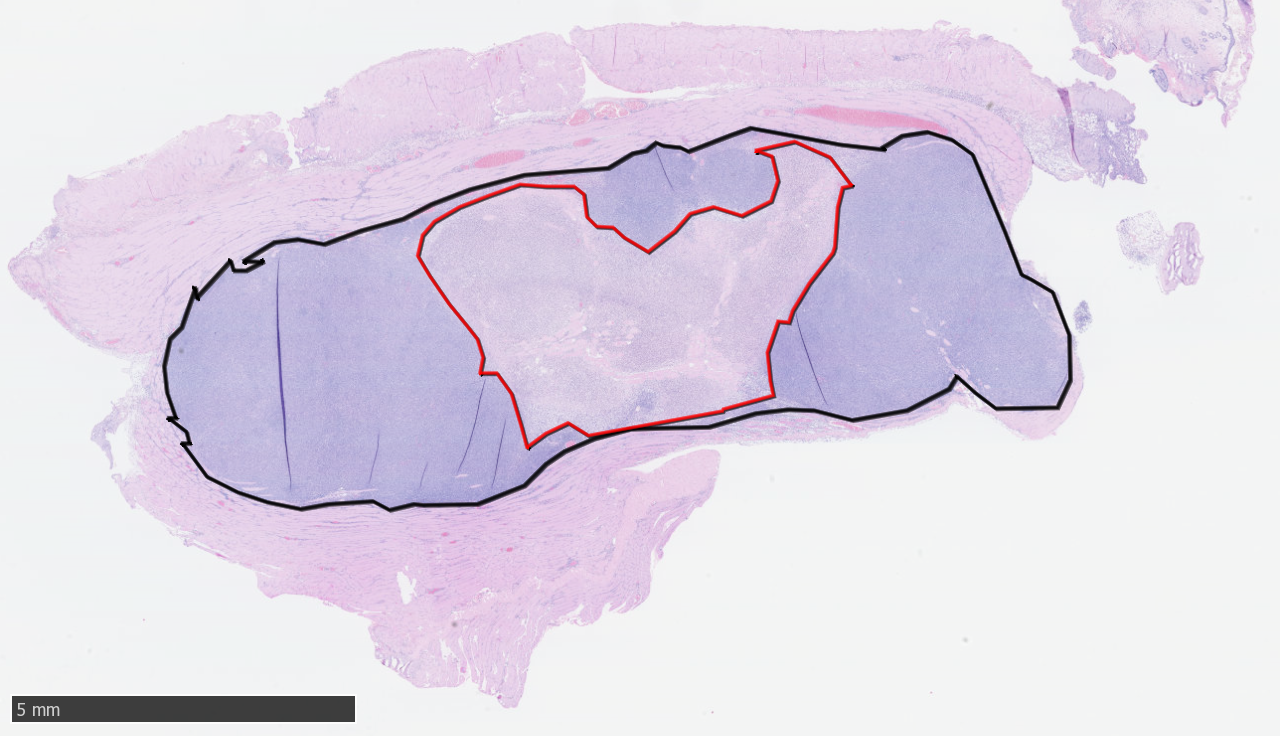

Supplement: Supplementary file 6 — Source Data for Figure 3 [file EMMM-12-e10979-s005.zip › Figure 3/ Figure 3C ILP-TNF:Mel:SM .tif]
